# Characterization of vB_ValM_PVA8, a broad-host-range bacteriophage infecting *Vibrio alginolyticus* and *Vibrio parahaemolyticus*

**DOI:** 10.3389/fmicb.2023.1105924

**Published:** 2023-05-12

**Authors:** Jingyun Fu, Ying Li, Lihong Zhao, Chunguang Wu, Zengguo He

**Affiliations:** ^1^College of Medicine and Pharmacy, Ocean University of China, Qingdao, China; ^2^Marine Biomedical Research Institute of Qingdao Co., Ltd., Qingdao, China; ^3^Qingdao Bioantai Biotechnology Co., Ltd., Qingdao, China

**Keywords:** phage therapy, *Vibrio alginolyticus*, *Vibrio parahaemolyticus*, *Vibrio* phage, *Vibrio* control, shrimp

## Abstract

Phage therapy was taken as an alternative strategy to antibiotics in shrimp farming for the control of *Vibrio* species of *Vibrio parahaemolyticus* and *Vibrio alginolyticus,* which cause substantial mortality and significant economic losses. In this study, a new *Vibrio* phage vB_ValM_PVA8 (PVA8), which could efficiently infect pathogenic isolates of *V. alginolyticus* and *V. parahaemolyticus*, was isolated from sewage water and characterized by microbiological and *in silico* genomic analyses. The phage was characterized to be a member of the *Straboviridae* family with elongated head and contractile tail by transmission electron microscopy. Genome sequencing showed that PVA8 had a 246,348-bp double-stranded DNA genome with a G + C content of 42.6%. It harbored totally 388 putative open reading frames (ORFs), among them 92 (23.71%) assigned to functional genes. Up to 27 transfer RNA (tRNA) genes were found in the genome, and the genes for virulence, antibiotic resistance, and lysogeny were not detected. NCBI genomic blasting results and the phylogenetic analysis based on the sequences of the large terminase subunits and the DNA polymerase indicated that PVA8 shared considerable similarity with *Vibrio* phage V09 and bacteriophage KVP40. The phage had a latent period of 20 min and a burst size of 309 PFUs/infected cell with the host *V. alginolyticus*, and it was stable over a broad pH range (4.0–11.0) and a wide temperature span (−80°C to 60°C), respectively, which may benefit its feasibility for phage therapy. In addition, it had the minimum multiplicity of infection (MOI) of 0.0000001, which revealed its strong multiplication capacity. The shrimp cultivation lab trials demonstrated that PVA8 could be applied in treating pathogenic *V. parahaemolyticus* infection disease of shrimp with a survival rate of 88.89% comparing to that of 34.43% in the infected group, and the pond application trails confirmed that the implementation of PVA8 could rapidly yet effectively reduce the level of the *Vibrio*. Taken together, PVA8 may be potential to be explored as a promising biological agent for *Vibrio* control in aquaculture farming industry.

## 1. Introduction

Facilitated with the adoption of high stocking density farming practice, the global aquaculture industry has experienced a rapid development worldwide in the past 30 years. Unfortunately, the high stocking density mode is often accompanied by the incidence of pathogenic bacteria caused diseases which results in severe economic losses. Family *Vibrionaceae* is one of the major sources of the pathogens, which are normal ubiquitous bacteria of the aquatic system and responsible for most of the bacterial outbreaks recorded across the countries (Seghal Kiran et al., 2016). Among them, a panel of species poses as pathogens causing disease in commercially crustacean, shellfish, and fish (Thompson et al., 2004). *Vibrio alginolyticus* is one of the most famous disease-causing *Vibrio* species that can infect a variety of aquatic animals, and it has been implicated in several types of mass mortality cases in major aquaculture species, such as fish, mollusks, and crustaceans (Letchumanan et al., 2016). The bacterium is also pathogenic to humans, causing skin and ear infections as well as acute gastroenteritis (Tan et al., 2015). *Vibrio parahaemolyticus* is another important disease-causing *Vibrio* species, which is pathogenic to both humans and marine animals (Su and Liu, 2007). It has been considered as a major concern for human health because it often causes acute gastroenteritis when people consume raw seafoods contaminated with this bad bug (Su and Liu, 2007; Iwamoto et al., 2010). Strikingly, *V. parahaemolyticus* can cause acute hepatopancreatic necrosis disease (AHPND) or early mortality syndrome (EMS), which is one of the most serious diseases in shrimp aquaculture which has led to devasting economic losses in the shrimp industry worldwide (Kalatzis et al., 2018).

Routinely, disinfectants and antibiotics are widely used in farming practice to prevent *Vibrio*. But these chemicals are becoming ineffective due to the emergence of the drug resistant by *Vibrio*. Moreover, the use of these chemicals has brought a series of negative effects, such as chemical pollution, the dysbiosis due to accumulated antibiotic residues, and the emergence of resistant bacteria derived in seawater (Kalatzis et al., 2018; Kosznik-Kwaśnicka et al., 2019; Matamp and Bhat, 2019). Therefore, alternative strategies to combat pathogens in aquaculture are in need (Weber-Dabrowska et al., 2016).

Bacteriophages, or phages for short, are viruses that can infect and kill their specific host bacteria. They are highly abundant in marine environments (Rasmussen et al., 2019). As a natural biocontrol method, phage therapy is characterized by high efficiency, specificity, and environmental friendliness. With the emerging of antibiotic resistance, phage therapy has been revitalized and considered as a potential alternative to antibiotic therapy in coping with bacterial infections (Choudhury et al., 2016; Sieiro et al., 2020). Nowadays, phage therapy has been explored in medicine (Onsea et al., 2019; Ooi et al., 2019), agriculture (Svircev et al., 2018; Kahn et al., 2019), food (Clavijo et al., 2019; Vaz et al., 2020), and environmental fields (Ayyaru et al., 2018; Sun et al., 2019). Similarly, phage therapy has great potential in aquaculture against pathogenic bacteria (Le and Kurtböke, 2019; Le et al., 2018). Recently, many successful cases of phage therapy for preventive and therapeutic purposes in aquaculture have been reported, using phages targeting *Vibrio* species including *V. parahaemolyticus* (Wong et al., 2019; Tan et al., 2021), *V. alginolyticus* (Li et al., 2019; Chen et al., 2020), *Vibrio harveyi* (Misol et al., 2020; Wu et al., 2021), *Vibrio vulnificus* (Kim et al., 2021), *Vibrio anguillarum* (Mauritzen et al., 2020), *Vibrio campbellii* (Lomeli-Ortega et al., 2021), *Vibrio mediterranean* (Xu et al., 2021) and other species including *Pseudomonas aeruginosa* (Cafora et al., 2019), *Aeromonas hydrophila* (Le et al., 2018; Pan et al., 2022; SumeetRai et al., 2023), and *Flavobacterium columnare* and other fish pathogens.

For phage to serve as biocontrol agent against *Vibrio*, a full characterization of their genomes is needed so that the presence of potentially harmful genes related to virulence or antibiotic resistance are ruled out as well as their lytic or temperate nature are revealed (Kokkari et al., 2018). To date, approximately hundreds of phages infecting *Vibrio* hosts have been isolated, completely sequenced, and deposited in the National Center for Biotechnology Information (NCBI) genome database. Most of them are phages infecting *V. parahaemolyticus*, *V. cholerae*, and *V. vulnificus*. Until recently, the complete genomes of several *V. alginolyticus* phages had been reported, including those myovirus-like phages [VP01 (Sasikala and Srinivasan, 2016), PVA1 (Cui et al., 2014), ValKK3 (Lal et al., 2016), and PVA23 (Fu et al., 2022)], those podovirus-like phages [IM271 (Li et al., 2019), VEN (Kokkari et al., 2018), ϕA318 (Lin et al., 2012), and vB_ValP_VA-RY-3 (Ren et al., 2022)], and those siphovirus-like phages [ValSw3-3 (Chen et al., 2020) and Artemius (Droubogiannis et al., 2022)]. Adding to this list, some potent *V. alginolyticus* phages have been documented, however, the complete genomic information of them still remain unavailable (Sasikala and Srinivasan, 2016; Le et al., 2020). To enlarge the arsenals for *Vibrio* control, more fully characterized phages are in need.

Previously, a potent *Vibrio* phage vB_ValM_PVA8 (PVA8) was isolated from local sewage samples. It exhibited decent efficiency in lysing *V. alginolyticus* and *V. parahaemolyticus*. In current study, we reported the phenotypical determination and the genomic characterization of PVA8, together with the evaluation of its application against pathogens including *V. alginolyticus* and *V. parahaemolyticus*.

## 2. Materials and methods

### 2.1. Bacteria strains and growth conditions

*Vibrio parahaemolyticus* ATCC 17802 purchased from Guangdong Microbial Culture Collection Center. Other strains used, including *V. alginolyticus* (Table 1), *V. parahaemolyticus* (Table 2) and other species of *Vibrio* and non-*Vibrio* strains (Table 3), were from the stocks of our lab. All *Vibrio* strains were isolated from seawater and hepatopancreas of diseased shrimps collected from different regions of China using the methods as reported (George et al., 2005). The *Vibrio* strains were stored at −80°C in 2216E liquid medium (Qingdao Hope Bio-Technology Co., China) with 30% glycerol. They were sub-cultured from −80°C stocks onto 2216E agar plates (2216E with 2% agar) and cultivated at 37°C for a minimum of 24 h. Unless otherwise specified, the *Vibrio* strains were cultured under standard conditions: liquid 2216E, 220 rpm aeration, and at 37°C.

**Table 1:** Host ranges of phage PVA8 to the *Vibrio alginolyticus* strains.

Number	Strains	Isolation source	*AspA*	*fur*	*tlh*	*tdh*	*trh*	Lysis
1	VA0	Ningbo, Zhejiang	+	+	+	−	−	+++
2	VA1	Ningbo, Zhejiang	+	+	+	−	−	+++
3	VA3	Nantong, Jiangsu	+	+	+	−	−	++
4	VA5	Rudong, Jiangsu	+	+	+	+	+	+
5	VA6	Rudong, Jiangsu	+	+	+	−	−	+++
6	VA7	Rudong, Jiangsu	+	+	+	−	−	+++
7	VA8	Ningbo, Zhejiang	+	+	+	−	−	+
8	VA9	Ningbo, Zhejiang	+	+	+	−	−	+++
9	VA10	Ningbo, Zhejiang	+	+	+	−	−	+++
10	VA11	Ningbo, Zhejiang	+	+	+	−	−	+++
11	VA12	Ningbo, Zhejiang	+	+	+	−	−	−
12	VA13	Nantong, Jiangsu	+	+	+	+	+	+
13	VA14	Nantong, Jiangsu	+	+	+	−	−	++
14	VA15	Nantong, Jiangsu	+	+	+	+	+	+++
15	VA16	Ningbo, Zhejiang	+	+	−	−	+	−
16	VA17	Ningbo, Zhejiang	+	+	+	+	+	++
17	VA18	Ningbo, Zhejiang	+	+	+	−	+	+
18	VA19	Nantong, Jiangsu	+	+	+	+	+	++
19	VA20	Nantong, Jiangsu	+	+	+	+	−	++
20	VA21	Nantong, Jiangsu	+	+	+	−	−	−
21	VA22	Nantong, Jiangsu	+	+	−	−	−	++
22	VA23	Qingdao, Shandong	+	+	+	+	−	++
23	VA24	Qingdao, Shandong	+	+	+	−	+	+++
24	VA25	Qingdao, Shandong	+	+	+	−	−	++
25	VA26	Qingdao, Shandong	+	+	+	−	−	+++
26	VA27	Qingdao, Shandong	+	+	+	−	+	+
27	VA28	Qingdao, Shandong	+	+	+	−	+	++
28	VA29	Qingdao, Shandong	+	+	+	+	−	−
29	VA30	Qingdao, Shandong	+	+	+	+	−	+
30	VA31	Rizhao, Shandong	+	+	+	−	−	+
31	VA32	Rizhao, Shandong	+	+	+	−	+	+
32	VA33	Rizhao, Shandong	+	+	+	−	−	+
33	VA34	Rizhao, Shandong	+	+	−	−	+	−
34	VA35	Rizhao, Shandong	+	+	+	−	+	+
35	VA36	Rizhao, Shandong	+	+	+	+	−	−
36	VA37	Rizhao, Shandong	+	+	+	−	+	+
37	VA38	Nanning, Guangxi	+	+	+	−	+	++
38	VA39	Nanning, Guangxi	+	+	+	−	−	+
39	VA40	Nanning, Guangxi	+	+	+	+	−	−
40	VA41	Nanning, Guangxi	+	+	+	−	−	+

+++, clear plaques; ++, plaques with slightly turbidity; +, plaques with heavy turbidity; −, no plaques formed. The growth conditions were all 2216E agar and broth at 37°C.

**Table 2:** Host ranges of phage PVA8 to the *Vibrio parahaemolyticus* strains.

Number	Strains	Isolation source	*toxR*	*tlh*	*trh*	*tdh*	*PirA*	Lysis
1	17,802	ATCC	+	+	+	−	−	−
2	VP1	Ningbo, Zhejiang	+	+	−	−	−	−
3	VP2	Ningbo, Zhejiang	+	+	−	−	−	+
4	VP3	Nantong, Jiangsu	+	+	−	−	−	+++
5	VP4	Rudong, Jiangsu	+	+	−	−	−	+
6	VP5	Rudong, Jiangsu	+	+	−	−	+	+
7	VP6	Rudong, Jiangsu	+	+	−	−	−	+
8	VP7	Ningbo, Zhejiang	+	+	−	−	+	+
9	VP8	Ningbo, Zhejiang	+	+	−	−	+	+++
10	VP9	Ningbo, Zhejiang	+	+	−	−	−	+
11	VP10	Ningbo, Zhejiang	+	+	−	−	−	+
12	VP11	Ningbo, Zhejiang	+	+	−	−	−	+
13	VP12	Nantong, Jiangsu	+	+	−	−	−	−
14	VP13	Nantong, Jiangsu	+	+	−	−	−	++
15	VP15	Nantong, Jiangsu	+	+	−	−	+	+
16	VP16	Ningbo, Zhejiang	+	+	−	−	−	+
17	VP19	Ningbo, Zhejiang	+	+	−	−	−	−
18	VP21	Ningbo, Zhejiang	+	+	−	−	−	+
19	VP22	Nantong, Jiangsu	+	+	−	−	−	+
20	VP27	Nantong, Jiangsu	+	+	−	−	−	−
21	VP32	Nantong, Jiangsu	+	+	−	−	−	++
22	VP33	Qingdao, Shandong	+	+	−	−	−	−
23	VP34	Qingdao, Shandong	+	+	−	−	−	+
24	VP35	Qingdao, Shandong	+	+	−	−	−	++
25	VP36	Qingdao, Shandong	+	+	−	−	−	+
26	VP37	Qingdao, Shandong	+	+	−	−	−	−
27	VP38	Qingdao, Shandong	+	+	−	−	−	+++
28	VP39	Qingdao, Shandong	+	+	−	−	−	−

+++, clear plaques; ++, plaques with slightly turbidity; +, plaques with heavy turbidity; −, no plaques formed. The growth conditions were all 2216E agar and broth at 37°C.

**Table 3:** Host ranges of phage PVA8 to other bacterial species of *Vibrio* and non-*Vibrio* strains.

Bacterial species	Medium	Total number of strains	Lysis number of strains
*Vibrio cholerae*	2216E	3	3
*Vibrio vulnificus*	2216E	3	0
*Vibrio fluvius*	2216E	4	0
*Vibrio harveyi*	2216E	2	0
*Vibrio campbellii*	2216E	3	0
*Vibrio mimicus*	2216E	5	4
*Vibrio neocaledonicus*	2216E	10	6
*Vibrio owensii*	2216E	7	3
*Vibrio antiquaries*	2216E	5	2
*Bacillus subtilis*	LB	4	0
*Bacillus licheniformis*	LB	2	0
*Lactic acid bacteria*	MRS	3	0
*Photosynthetic bacteria*	*Photosynthetic bacteria* medium	2	0
*Saccharomyces bacteria*	PDA	3	0

### 2.2. Detection of virulence genes of the *Vibrio alginolyticus* and *Vibrio parahaemolyticus* strains

A panel of common virulence genes including alkaline serine protease gene (*AspA*), ferric uptake system gene (*fur*), and hemolysin genes (*tlh*, *tdh,* and *trh*) of *V. alginolyticus* and photorhabdus insect related toxin A gene (*PirA*), virulence-regulating genes (*toxR*), and hemolysin genes (*tlh*, *tdh,* and *trh*) of *V. parahaemolyticus* were detected by PCR assay (Kahla-Nakbi et al., 2006; Fethi et al., 2011; Table 4). The PCR amplification was performed under the following conditions: initial denaturation at 94°C for 4 min, followed by 35 cycles of 94°C for 1 min, 60°C for 1 min, and 72°C for 1.5 min; and a final extension at 72°C for 8 min. Amplified PCR products were visualized on a 1% agarose gel stained with ethidium bromide, running at 90 V for 25 min. The presence of virulence genes was confirmed by gel electrophoresis that showing the corresponding target band by the gel imaging system after repeating the PCR three times.

**Table 4:** Primers used in this study for amplification of virulence genes of the *Vibrio alginolyticus* and *Vibrio parahaemolyticus* strains.

*Vibrio* species	Gene	Primer	Size (bp)	References
*V. alginolyticus*	*AspA*	F: GCATGGTACTCACGTAGCGG	146	Xiong et al. (2014)
		R: CTTTCACAAGACCAGAAGAGTAACC		
	*fur*	F: ATTAACCCTTTGAAGTTCGTGG	111	Xiong et al. (2014)
		R: TGACATATACTTTCCCGTTGGATC		
	*tlh*	F: CGAACGAGAACGCAGACATT	108	Xiong et al. (2014)
		R: CTTTGTTGATTTGATCTGGCTG		
	*tdh*	F: ATAAAGACTATACAATGGCAGCGG	138	Xiong et al. (2014)
		R: GAATAGAACCTTCATCTTCACCAAC		
	*trh*	F: GCCTTTCAACGGTCTTCACAA	179	Xiong et al. (2014)
		R: TAACAAACATATGCCCATTTCCG		
*V. parahaemolyticus*	*PirA*	F: ATGAGTAACAATATAAAACATGAAAC	336	Thadtapong et al. (2020)
		R: GTGGTAATAGATTGTACAGAA		
	*toxR*	F: TCATTTGTACTGTTGAACGCCT	374	Wang et al. (2014)
		R: AATAGAAGGCARCCAGTTGTT		
	*tlh*	F: CGAAGAGCCAACCTTATCA	759	Lin et al. (2016)
		R: TCCGTCAAACGAATCAGTG		
	*tdh*	F: CTTCCATCTGTCCCTTTTCC	244	Lin et al. (2016)
		R: CCGCTGCCATTGTATAGTCT		
	*trh*	F: TTTCCTTCTCCTGGTTCCG	418	Lin et al. (2016)
		R: TATGTCCATTGCCGCTCT		

### 2.3. Phage isolation, purification, and propagation

Isolation of phages was conducted using the conventional double-layer agar method (Chen et al., 2019). All the strains of *V. alginolyticus* and *V. parahaemolyticus* mentioned above were used as hosts to isolate phages. Sewage samples were collected from a large-scale aquatic market in Qingdao, China. First, sewage water (20 mL) was centrifuged at 8,000 × *g* for 10 min, and filtered through a 0.22 μm membrane to get rid of bacteria. Next, 5 mL of the filtrate and 5 mL of *Vibrio* culture in logarithmic phase (10^7^ CFU/mL) were added to 50 mL of sterilized 2216E medium. The mixture was incubated at 37°C with orbital shaking for 6 h to enrich possible phages, then it was centrifuged at 8,000 × *g* for 10 min and filtered through a 0.22 μm membrane to remove the remaining bacteria. 100 μL of the filtrate, mixed with 100 μL of *Vibrio* culture, was poured into 5 mL of molten soft 2216E upper agar (2216E liquid medium with 0.7% agar) and then poured onto the surface of a prepared and cooled 2216E lower plate (2216E liquid medium with 1.5% agar). Phage plaques appearance was observed after the overnight incubation at 37°C.

A single plaque was picked into 1 mL of sterilized PBS buffer (8 g NaCl, 0.2 g KCL, 1.44 g Na_2_HPO_4_ 12H_2_O, 0.24 g KH_2_PO_4_, 0.11 g CaCl_2_, adding water to 1,000 mL and adjusting pH to 7.2 with Na_2_HPO_4_ 12H_2_O or KH_2_PO_4_) and bathed in water at 40°C for 30 min to harvest phages. Then the phage-containing PBS buffer was centrifuged at 12,000 × *g* for 5 min and filtered through a 0.22 μm membrane. This filtrate was diluted at a 10-fold gradient with PBS buffer to a suitable multiple which could obtain single plaques on the double-agar plate. The single-plaque isolation process by the double-layer agar method was repeated four more times until plaques of uniform size, shape, and clarity were obtained.

The lysate of the pure single plaque in 100 μL PBS was mixed with equal amount of fresh culture of the host strain, and then was added into 5 mL of 2216E tube and incubated at 37°C with shaking at 220 rpm to getting phage proliferation solution. Cell debris of the solution was removed by centrifugation and filtration. The bacteriophage titer of the filtrate was determined using the double-layer agar method and expressed as plaque-forming units (PFU) per mL filtrate. Finally, the proliferation solution was stored at 4°C for further experiments.

### 2.4. Phage host ranges determination

Phage host ranges was determined by the method of standard spot tests assay (Feng et al., 2021). All the strains of *V. alginolyticus* and *V. parahaemolyticus* mentioned above were tested. Briefly, 100 μL of each bacterium in logarithmic phase (10^7^ CFU/mL) was added to 5 mL of molten soft 2216E upper agar and then poured onto the surface of a prepared and cooled 2216E lower plate. 2 μL of phage stock (10^9^ PFU/mL) was spotted onto the lawn and incubated at 37°C for 12 h. Finally, the spots observed were classified into four categories based on their clarity, which were clear, slight turbidity, heavy turbidity, and unresponsive, respectively. The test was repeated three times to obtain reliable results.

In addition, a panel of other bacterial species of *Vibrio* and non-*Vibrio*, commonly found in pond waters, were also tested to determine the host ranges of the phage using the same method.

### 2.5. Transmission electron microscopy

The morphology of the phage was characterized by using transmission electron microscopy (Chen et al., 2019). Phage particles were precipitated with 10% polyethylene glycol 8,000 (PEG 8000) at 4°C overnight, centrifuged at 10,000 × *g* for 15 min, and subsequently suspended in PBS buffer. One drop of the concentrated phage particles was spotted onto the surface of carbon grids for 15 min and then negatively stained with 2% phosphotungstic acid for 10 min. Then the phage was observed using a Hitachi H-7700 biological transmission electron microscope (Hitachi High-Technologies Corporation, Tokyo, Japan) at an accelerating voltage of 80 kV. It was classified and identified on the basis of International Committee on Taxonomy of Viruses (ICTV) guidelines.

### 2.6. Optimum adsorption time, multiplicity of infection, and one-step growth curve experiments

The optimum adsorption time of the phage to the host bacteria was determined using methods described previously (Qi et al., 2020). Briefly, 50 μL of phage stock (10^9^ PFU/mL) and 50 ml of log-phase *V. alginolyticus* VA10 culture (10^7^ CFU/mL) were mixed evenly in the test tube to achieve an MOI at 0.1, then followed by immediate incubation at 37°C with shaking. This moment was defined as *t* = 0 min, and 1 mL of the mixture was collected at every 1 min before 10 min and every 5 min after 10 min until 30 min (1 tube per time point). The titer of phage particles was conducted using the double-layer agar method. All experiments were performed in triplicate. The percentage of free phage particles was calculated by the ratio of the average number of phage plagues at each time point to the average number of phage plagues at 0 min. The percentage of adsorbed phage particles was obtained by 1 minus the percentage of free phage particles. The time of producing the highest percentage of adsorbed phage particles is the optimum adsorption time of the phage to the host.

The optimal MOI for the phage was determined using methods described previously (Xi et al., 2019). Briefly, phage stock (10^9^ PFU/mL) were added to 5 mL of log-phase *V. alginolyticus* VA10 culture (10^7^ CFU/mL) to achieve an MOI of 0.0000001, 0.000001, 0.00001, 0.0001, 0.001, 0.01, 0.1, 1, 10, and 100, respectively, and then incubated at 37°C, 220 rpm for 6 h. Culture supernatant was then filtered through a 0.22-μm filter, and the titer of the phage in the supernatant was using the double-layer agar method. The experiment was carried out in triplicate. The MOI resulting in the highest phage titer was considered the optimal MOI of the phage.

In this study, the one-step growth curve experiment was conducted as previously described by Lee et al. (2016) with some modifications (Lee et al., 2016). First, the phage stock (10^9^ PFU/mL) was mixed with the host *V. alginolyticus* VA10 culture in logarithmic phase (10^7^ CFU/mL) at a MOI of 0.1 and incubated at 37°C for 10 min to allow the phage to adsorb the bacteria. After incubation, the broth was centrifuged at 12,000 × *g* for 30 s to remove unabsorbed free phage. Next, the pellets were washed with 2216E for twice at 37°C, and the suspension was transferred to 20 ml of 2216E followed by immediate incubation at 37°C with shaking. This moment was defined as *t* = 0 s, and 200-μL sample was collected every 10 min up to 180 min. The titer of phage particles was conducted using the double-layer agar method. All experiments were performed in triplicate. The burst size of phage was calculated as the ratio of the final count of liberated phage particles to the initial count of infected bacterial cells.

### 2.7. Thermal and pH stability assays

The thermal stability assay of the phage was performed as previously described (Shahin and Bouzari, 2017). Phage stock (10^9^ PFU/mL) was incubated at different temperatures of 40°C, 50°C, 60°C, 70°C, and 80°C, respectively. Aliquots of 200-μL were taken at 20, 40, and 60 min during the incubation at each temperature, respectively. In addition, the phage lysate is kept at −80°C for a long time, and aliquots of 200-μL are taken every one month to test the activity. To evaluate the pH stability of the phage, phage stock (10^9^ PFU/mL) was treated with 10 ml PBS buffers with varied pH values (2–13, adjusted with NaOH or HCl) at 37°C for 2 h. Phage titers were determined using the double-layer agar method. The experiments were conducted in triplicate.

### 2.8. Chloroform and ultraviolet sensitivity assays

To determine the chloroform sensitivity of the phage, PVA8 stock (5 ml, 10^9^ PFU/mL) was mixed with chloroform to get final concentration at 1%, 2%, 3%, 4%, and 5%, respectively, and the mix was incubated at 37°C for 30 min by shaking. After incubation, 200-μL aliquots was taken for phage titer measuring. To determine the impact of Ultraviolet (UV) irradiation, phage stock (10^9^ PFU/mL) was exposed to UV irradiation (20 W, 30 cm) and phage tittering was carried out at irregular intervals before 3 min and every 3 min after 3 min until 30 min. The experiments were conducted in triplicate.

### 2.9. Phage lytic ability *in vitro* assay

The ability of the phage to lyse the *V. alginolyticus* cells was determined in *in vitro* dose-dependent assay. The host *V. alginolyticus* VA10 culture in logarithmic phase (10^7^ CFU/mL) was mixed with a 10-fold serial dilution of the phage stock (10^9^ PFU/mL) to make MOIs at 0.1, 1, and 10, respectively. The mix was incubated at 37°C for 300 min with orbital shaking. Host culture untouched with the phage was served as the control. Aliquots were collected at 10-min intervals and the optical density (OD_600_) was measured and recorded. The experiment was conducted in triplicate.

### 2.10. Phage bacterial phage-insensitive mutation frequency assay

To determine the bacterial phage-insensitive mutation frequency (BIMF), the phage was used to lyse the host *V. alginolyticus* VA10 culture for resistance observation by following the method previously described (Lu et al., 2003; Gutierrez et al., 2015). The fresh cultures of the host in logarithmic phase (10^7^ CFU/mL, 100 μL) were used to coculture with phage stock (10^9^ PFU/mL, 100 μL) at 37°C for 10 min, the host bacterium without phage was used as the control. After serial dilution, the mixture was coated on TCBS plates and incubated at 37°C for 18 h, and the number of phage-resistant colonies in the plate was recorded (the number of assumed insensitive colonies). Then all the resistant colonies were picked for 2216E broth, and tolerance to the phage was confirmed by the spot assay method mentioned above. The number of colonies without lysis circle was recorded (the number of determined insensitive colonies). BIMF is the ratio of the number of determined insensitive colonies to the total number of colonies. The experiment was conducted in triplicate.

### 2.11. Phage DNA extraction, genome sequencing, and assembly

To increase our understanding of the phage at the genomic level, the phage DNA was extracted as previously described (Chen et al., 2019). First, phage particles were treated with DNase I and RNase A (New England BioLab, England) followed by incubating at 37°C for 30 min to remove any exogenous host genomic DNA and RNA. Then phage PVA8 genomic DNA was extracted using the λ Bacteriophage Genomic DNA Rapid Extracted Kit (Shanghai Yuanye Biotechnology Co., Ltd., China) following the manufacturer^’^s instructions. The purified phage PVA8 DNA was performed on an Illumina NovaSeq sequencing platform (Qingdao Weilai Biotechnology Co., Ltd., China). The filtered reads were assembled using SOAPdenovo by the default parameters. The complete genome of phage PVA8 was finished and then manually inspected.

### 2.12. Genome analysis and phylogenetic analysis

All open reading frames (ORFs) of the complete phage genome sequence are predicted by the program GeneMarkS (Besemer and Borodovsky, 2005). Annotation and functional analysis of all predicted ORFs were conducted using Subsystem Technology (RAST, https://rast.nmpdr.org/rast.cgi). BLASTp algorithm (https://blast.ncbi.nlm.nih.gov/Blast.cgi, protein–protein BLAST) against the non-redundant (nr) protein database of the National Center for Biotechnology Information [NCBI; (Song et al., 2009)] was used for further verification of the predicted proteins. The genome circle map was drawn by CGView server (Grant and Stothard, 2008). The genome sequences of 11 other closely related homologous phages were downloaded from NCBI database, and similarities between the genomic sequences were determined using the average nucleotide identity MUMmer (ANIm; Pritchard et al., 2016). Comparative whole-genome maps of phages were also visualized by CGView (Grant and Stothard, 2008). The putative tRNA-encoding genes were searched using tRNAscan-SE (Schattner et al., 2005).^1^ The genes for virulence and antibiotic resistance were detected by Virulence Factor Database (VFDB)^2^ and Antibiotic Resistance Database (ARDB),^3^ respectively (Underwood et al., 2005).

To determine the taxonomy of the phage, ML algorithm in FastTree software^4^ was used to construct phylogenetic tree based on the nucleotide sequence of the complete genome of phage, and the reliability of the branches was verified (bootstrap, 1,000 replications) after the establishment. Multiple-sequence alignment of the phage with the highest similar reference phages was performed using Easyfig 2.2 (Sullivan et al., 2011).

### 2.13. Phage treatment of shrimps infected with *Vibrio parahaemolyticus* VP8

To validate the therapeutic performance of the phage in controlling the pathogenic bacterial infections in practical applications, 450 healthy 60-day-old shrimp (*Lenaeus vannamei*; weight = 10 ± 0.5 g), cultured in 20 L glass tanks under appropriate conditions with aquatic water temperature maintaining at 25°C ± 1°C during the study, were equally divided into five groups. Each group contained 90 shrimps and was then divided equally into three parallel subgroups as replicates (30 shrimp per subgroup). The *V. parahaemolyticus* VP8 culture was poured into the glass tanks to challenge shrimps in the experiment. To prepare the cells of *V. parahaemolyticus* VP8, the culture in logarithmic phase was spined down and washed with sterile saline for three times and then resuspended to make a stock at a concentration of 2 × 10^9^ CFU/mL by measuring the optical density (OD_600_) and coating TCBS plates. The phage stock (2 × 10^10^ PFU/mL) was poured into the glass tanks to treat shrimps. Group 1 was set up without any treatment. Group 2 was supplemented with the phage stock at a final concentration of 2 × 10^7^ PFU/mL in the water but without the challenge of VP8. Group 3 was challenged with VP8 suspension at a final concentration of 2 × 10^6^ CFU/mL in the water for 2 days. Group 4 was treated with 10 mg/L doxycycline after a two-day challenge of VP8 at 2 × 10^6^ CFU/mL. Group 5 received the treatment of the phage stock at a final concentration of 2 × 10^7^ PFU/mL in the water after a 2-day challenge of VP8 at 2 × 10^6^ CFU/mL. The survival rate of the shrimp of each group was subsequently summarized after 7-day cultivation, and the status of shrimps was observed and monitored during the experiment.

### 2.14. Evaluation of phage application against *Vibrio* in shrimp farming plants

The experiment was made in a shrimp farming plant located in Yantai, China, which was farming for shrimps (*P. vannamei*, average weight = 6 g ± 0.5 g), kept in 30-m^3^ pond under appropriate conditions (water temperature 26°C ± 1°C, salinity 25‰). The pond water in this plant was changed by 30% every day. To control the propagation of pathogenic *Vibrio*, the pond was regularly added with agents, such as povidone-iodine, complex iodine, chlorine dioxide and probiotic products, etc., which were stopped at least 72 h before the phage tests.

The experiments contained four groups, and the *Vibrio* contents of the ponds in each group were detected by coating TCBS plates before the start. Group 1 was the blank control receiving no control agent. Group 2 was supplemented with an iodic disinfectant (found in market) at dose of 5 mg/L. Group 3 was supplemented with a probiotic product (Qingdao Bioantai Biological Technology Co., LTD, Implementation standard Q/370212BAAT001-2019) at dose of 5 mg/L. Group 4 was supplemented with the phage stock (10^10^ PFU/mL) with the MOI at 0.1. Each group of the tests repeated twice, with pond water sampled every 4 h and the *Vibrio* content checked.

### 2.15. Phage preservation and nucleotide sequence accession number

The phage was preserved in the China General Microbiological Culture Collection Center (Beijing, China) with CGMCC number of No.24098. The complete genome sequence of the phage has been submitted to the NCBI GenBank database under accession number of OQ164647.

### 2.16. Statistical analyses

Data were analyzed by one-way analysis of variance (ANOVA) using GraphPad Prism 8.0. Significant differences were determined using Tukey’s Multiple Comparison Test. Statistical significance was considered at the *p* < 0.05 level, and the results were expressed as mean ± standard deviation (SD).

## 3. Results

### 3.1. Profile of virulence genes of the *Vibrio alginolyticus* and *Vibrio parahaemolyticus* strains

**Figure 1:**
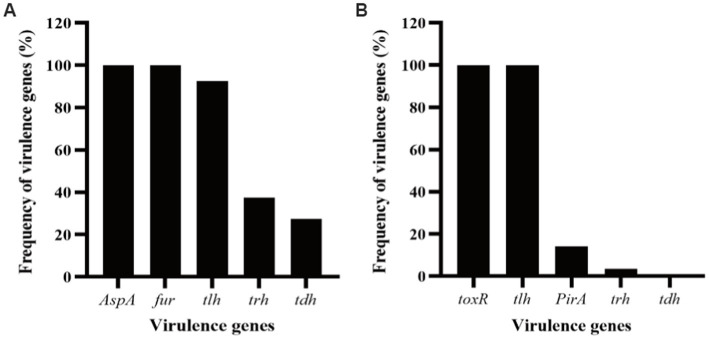
Frequency of virulence genes detected in the strains of *Vibrio alginolyticus* and *Vibrio parahaemolyticus.* **(A)** Five virulence genes, *AspA*, *fur*, *tlh*, *tdh,* and *trh*, were selected for PCR detection for the *V. alginolyticus* strains. **(B)** Five virulence genes, *toxR*, *PirA, tlh*, *tdh,* and *trh*, were selected for PCR detection for the *V. parahaemolyticus* strains.

In this work, up to 40 *V. alginolyticus* strains and 28 *V. parahaemolyticus* strains were used. In order to investigate the virulent features of these strains, a panel of virulence-associated genes including alkaline serine protease gene (*AspA*), ferric uptake system gene (*fur*), and hemolysin genes (*tlh, tdh,* and *trh*) of *V. alginolyticus* and photorhabdus insect related toxin A gene (*PirA*), virulence-regulating genes (*toxR*), and hemolysin genes (*tlh*, *tdh,* and *trh*) of *V. parahaemolyticus*, were checked by PCR assay. For the *V. alginolyticus* strains (Figure 1A), *AspA* and *fur* were the most prevalent genes with the detection frequencies of 100% (40/40), followed by *tlh* (92.5%, 37/40), *trh* (37.5%, 15/40), and *tdh* (27.5%, 11/40), respectively. All the *V. alginolyticus* strains carried virulent genes of *AspA* and *fur*, and the majority also carried the *tlh* gene. Among them, two strains, *V. alginolyticus* VA15 and VA17, carried all the five virulence genes. For the *V. parahaemolyticus* strains (Figure 1B), *toxR* and *tlh* were the most prevalent genes with the detection frequencies of 100% (28/28), followed by *PirA* (14.29%, 4/28) and *trh* (3.57%, 1/28), respectively, while the *tdh* gene had the detection frequency of 0% (0/28). Among them, four *V. parahaemolyticus* strains, VP5, VP7, VP8, and VP15, carried the *PirA* virulence gene, thus they were selected as the presentative strains for later phage lysis testing.

### 3.2. Phage isolation and host ranges determination

Sewage samples were collected from a large-scale aquatic market in Qingdao, China, and the *V. alginolyticus* and *V. parahaemolyticus* strains mentioned above were used to isolate and screen phages. Using these *Vibrio* strains as hosts, 124 bacteriophages were successfully isolated and purified from the sewage samples, including 63 *V. alginolyticus-*infecting bacteriophages (Supplementary Table S1) and 61 *V. parahaemolyticus*-infecting bacteriophages (Supplementary Table S2). These phages formed clear plaques on the double-layer plates after overnight incubation at 37°C. The host ranges of the isolated phages were then determined using these *Vibrio* strains by the method of spot tests. According to the results (Supplementary Tables S1, S2), a phage, named PVA8, with the VA10 as the host was screened for its high lysis rate of 82.5% (33/40) to the *V. alginolyticus* strains (Table 1). Then, we found that it could also effectively infect the *V. parahaemolyticus* strains with the lysis rates of 71.43% (20/28; Table 2).

In addition, the result of the host ranges of PVA8 using a panel of bacterial species of the other *Vibrio* and non-*Vibrio* is shown in Table 3. Except the *Vibrio* species of *V. alginolyticus* and *V. parahaemolyticus*, PVA8 could also infect other *Vibrio* species, but it did not exhibit any infectivity to bacterial species in genus other than *Vibrio*. This cross-species lytic ability and specificity for *Vibrio* species of phage PVA8 can be used to control different pathogenic *Vibrio* species and is a suitable choice for further study.

### 3.3. Phage identification based on morphological characteristics

**Figure 2:**
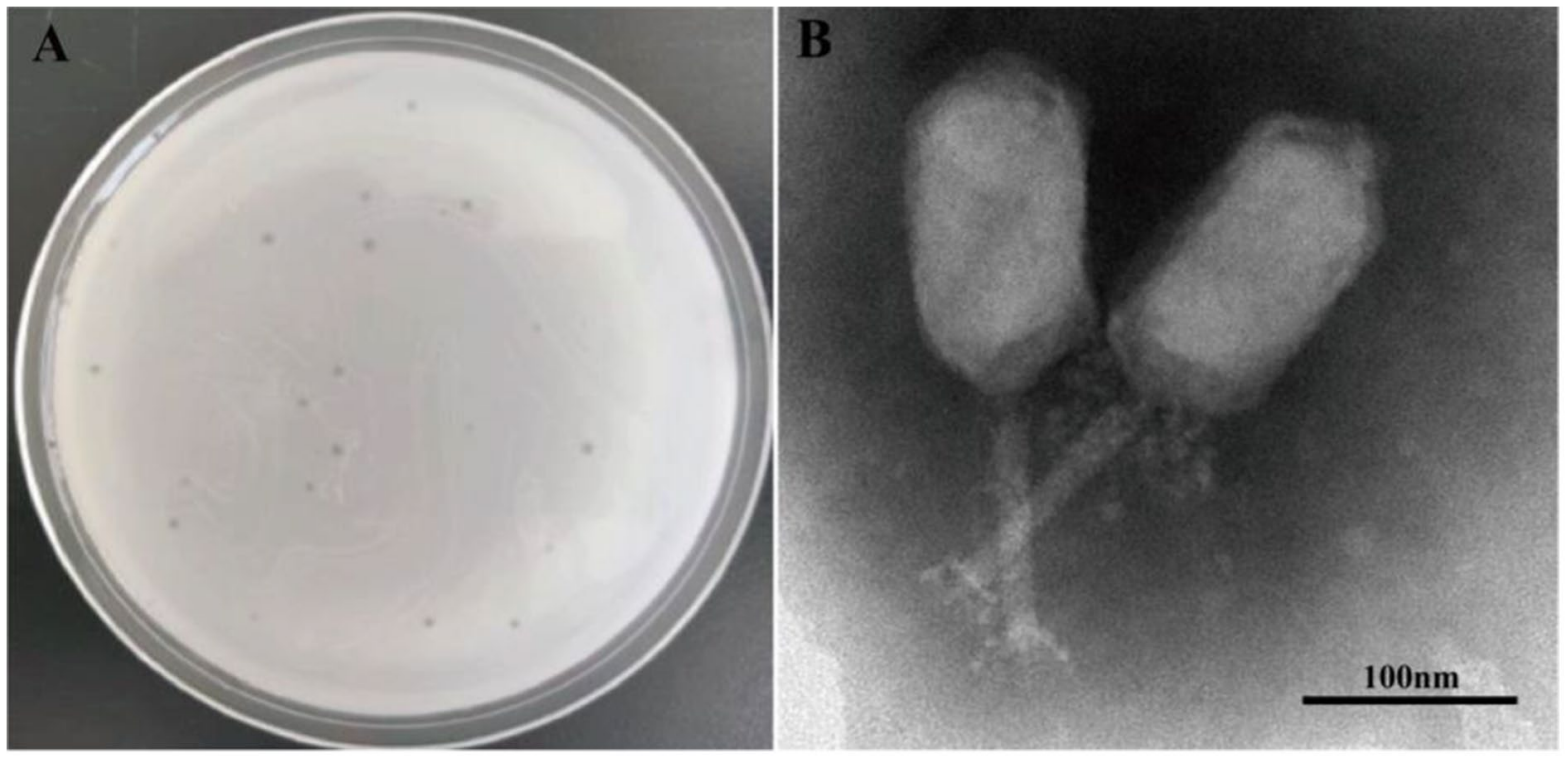
Phage plaques and transmission electron microscopy of phage PVA8. **(A)** Phage plagues formed on double-layer agar plate of *Vibro alginolyticus* VA10; **(B)** Transmission electron microscopy of PVA8, scale bar = 100 nm.

Phage PVA8 formed clear plaques on the lawn of the *V. alginolyticus* VA10, with an averaged diameter about 0.5 mm after 8 h incubation (Figure 2A). Purified phage was subjected to transmission electron microscopy observation. It was found that PVA8 had an elongated head (prolate capsid) with about 139.34 nm long and 71.72 nm wide and a contractile tail with about 112.45 nm long (Figure 2B). Based on the classification standards issued by International Committee on Taxonomy of Viruses (ICTV), PVA8 was identified as one member of the *Straboviridae* family.

### 3.4. Biological characteristics and stability features of phage PVA8

The percentage of adsorbed phages of the mixture reached to (88.5 ± 5.3)% at 8 min (Table 5), and after that the progeny of the phages began to release. Thus, the percentage of adsorbed phages could not be calculated since 9 min. The percentage of adsorbed phages of the mixture is the highest at 8 min, which is the optimum adsorption time of PVA8 to the host *V. alginolyticus* strain VA10.

**Table 5:** The result of the determination of optimal adsorption time of phage PVA8.

Time (min)	0	1	2	3	4	5	6	7	8	9
Percentage of adsorbed phages (%)	0	2.5 ± 2.3	13.8 ± 3.1	28.9 ± 2.4	42.3 ± 5.3	50.4 ± 6.8	65.4 ± 4.3	77.6 ± 3.3	88.5 ± 5.3	-

**Figure 3:**
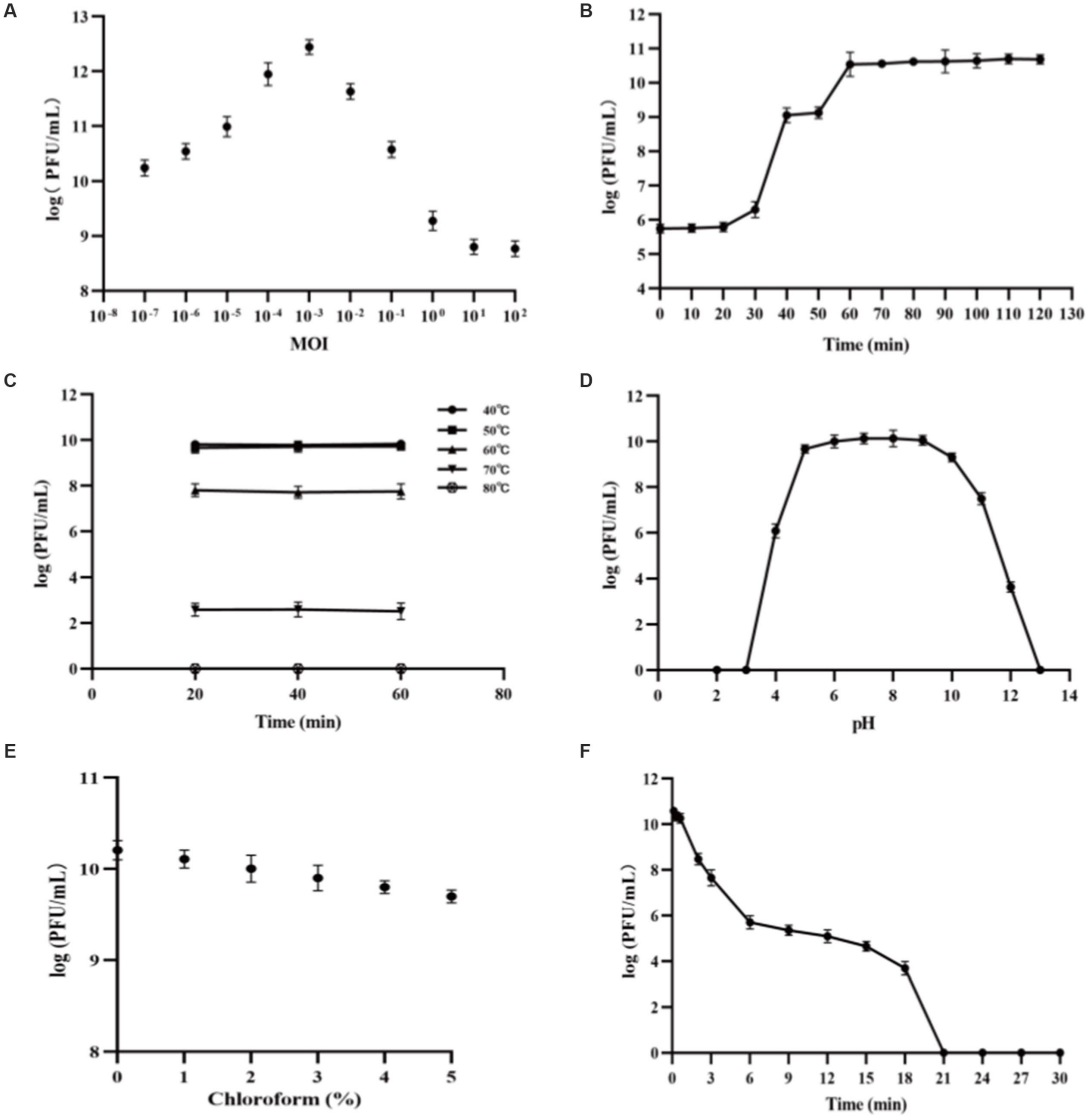
Biological characteristics and stability features of PVA8. **(A)** The MOI test of PVA8; **(B)** The one-step growth curve of PVA8; **(C)** The thermal stability test of PVA8; **(D)** The pH stability test of PVA8; **(E)** Chloroform sensitivity test of PVA8, and **(F)** Ultraviolet (UV) sensitivity test of PVA8.

With *V. alginolyticus* strain VA10 as the host, the highest titer of PVA8 amounted to 2.79 × 10^12^ PFU/mL at MOI of 0.001, and the lowest MOI of PVA8 was as low as 0.0000001, indicating that the phage could achieve high yield yet strong proliferative capacity (Figure 3A).

PVA8 was confirmed to have very strong lysis ability by the one-step growth curve determination, in which a short latent time (20 min) and a big burst size (309 PFUs/infected cell) were demonstrated, respectively (Figure 3B).

The thermal stability assay showed that PVA8 was fairly stable at temperatures below 60°C, but it was completely inactivated as temperature was at 80°C (Figure 3C). In addition, the titer of this phage remains stable for a long time in fridge at −80°C, indicating that it can be stored at this temperature for long time.

The pH stability test showed that PVA8 remained active over a broad pH range, from 4.0 to 12.0, for at least 2 h, but it was completely inactivated under harsh pH conditions (below pH 3.0 or above pH 13.0; Figure 3D).

As shown in Figure 3E, PVA8 was sensitive to the chloroform treatment by a concentration-dependent mode, which indicated the phage capsid protein might contain lipid-like structure.

The survival curve of PVA8 under UV irradiation was depicted in Figure 3F. The results demonstrated that PVA8 was sensitive to the UV irradiation, by following a time based PFU declination trend as observed. It was completely inactivated at 21 min after the treatment.

### 3.5. Lysis activity *in vitro* of phage PVA8

**Figure 4:**
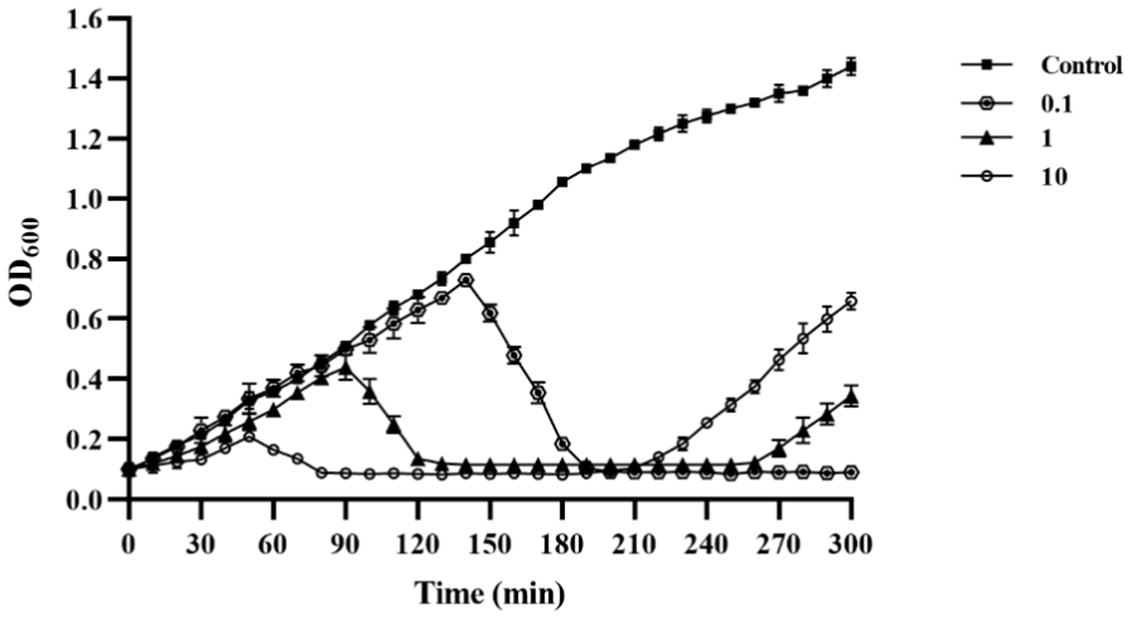
Lysis ability *in vitro* of phage PVA8 against the host organism VA10 at three different MOIs (10, 1, and 0.1).

*In vitro* tests were conducted to evaluate the lysis potential of phage PVA8, using *V. alginolyticus* VA10 strain as the host and by applying three MOIs at 0.1, 1, and 10, respectively (Figure 4). In the control, it was observed that the OD_600_ kept increasing and it amounted to 1.44 at 300 min. In contrast, the OD_600_ of all the phage treatments reduced significantly after the initial growth in the first few hours, indicating the lysis of the host cells caused by PVA8. It was also noticed that the lysis time was negatively correlated to the MOIs with a rough S-shape OD_600_ trend observed for each MOI, respectively. The OD_600_ rebounding might reflect the establishment of host resistance to the phage infection via some undefined escaping ways such as mutation or alteration.

### 3.6. Bacterial phage-insensitive mutation frequency of *Vibrio alginolyticus* VA10 to phage PVA8

In order to investigate the rebounding of the host strain, the bacterial phage-insensitive mutation frequency (BIMF) test was conducted. As shown in Table 6, the BIMF of *V. alginolyticus* VA10 to PVA8 was (6.39 ± 0.78) × 10^−7^, which was similar to those seen in previous reports (Gutierrez et al., 2015). This result reasoned why the rebounding of the host bacteria in the phage application experiment as observed, which implied that a certain mutation might occur when the target *Vibrio* strain was challenged with the phage. Thus, for the control of *V. alginolyticus* VA10, phage cocktails were suggested in practice to reduce the possible resistance derived due to mutations in the targeted *Vibrio*.

**Table 6:** The result of the BIMF of *Vibrio alginolyticus* VA10 to phage PVA8.

	Assumed insensitive colonies in plates	Determined insensitive colonies by standard spot test	Total colonies without phage	The BIMF
Phage group	6 ± 1	5 ± 1	-	(4.04 ± 0.56) × 10^−8^
Control group	-	-	(1.23 ± 0.03) × 10^8^	-

### 3.7. Genome sequencing, characterization, and analysis of phage PVA8

The complete genome of PVA8 was 246, 348 bp long, with 99.63% of the reads matched to the complete genome (3,909,018 out of 3,923,346).

**Figure 5:**
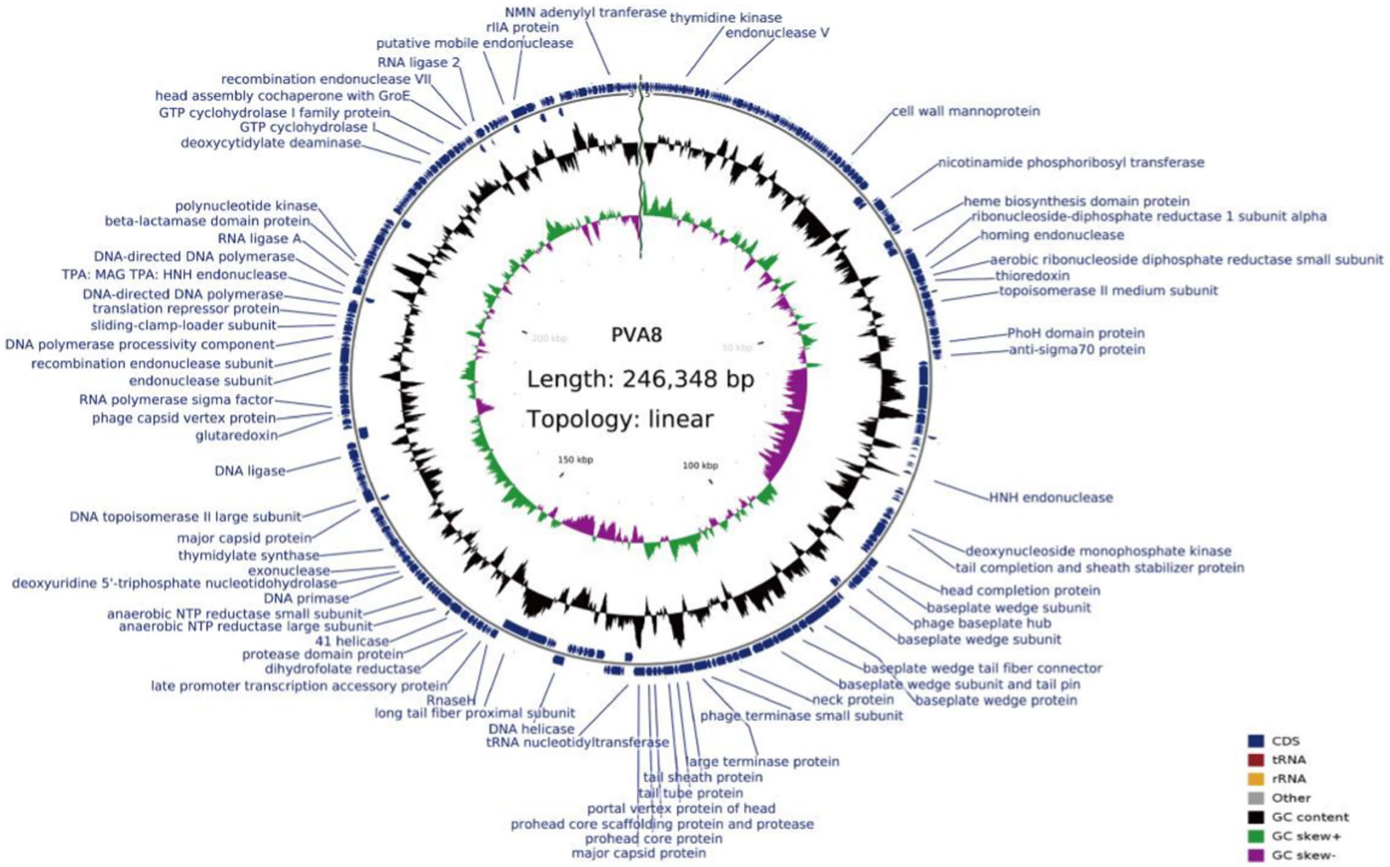
Genome circle map of phage PVA8. From inside to outside, the first circle represents the scale, the second circle represents GC Skew, the third circle represents G + C content, and the outermost circles represent the positions of CDs on the genome. In the second circle, the outward direction indicates that the GC-skew [(G − C)/(G + C)] is larger than zero, and the inward direction indicates that the GC-skew [(G − C)/(G + C)] is lower than zero. In the third circle, the outward direction indicates that the G + C content of this region is larger than the average G + C content of the whole genome, and the inward direction indicates that the G + C content of that region is lower than the average G + C content.

The genome circle map of phage PVA8 with NR annotations is shown in Figure 5. Sequencing analysis showed that the genome was a linear, double-stranded DNA molecule of 246, 348 bp in length with a GC content of 42.6%. It assigned 388 coding sequences (CDSs) with online RAST annotation. Among the 388 CDSs, 378 CDSs begin with the start codon ATG, only seven begin the start codon TTG, and three begin with the start codon GTG. Blastp analysis against the NCBI nr database showed that 92 ORFs (23.71%) were annotated to have corresponding functions. These ORFs related to the basic functions of phages can be identified and categorized, which covered proteins as DNA replication/modification, structure protein, packing protein, metabolism/regulation, and host lysis, respectively.

Several typical enzymes and proteins in DNA replication/modification module were predicted in the genome of PVA8, which are similar with those of other *Vibrio* phages. ORF207 was predicted to encode ribonuclease H, an enzyme that cleaves the RNA strand of an RNA/DNA hybrid (Ohyama et al., 2014), showing 100% identity to that of bacteriophage KVP40. DNA polymerase encoded by ORF290 has 97.17% homology to that of *Vibrio* phage V09, which can be used to fill DNA gaps generated during DNA repair, recombination, and replication. DNA helicase, an enzyme that utilizes ATP hydrolysis to separate the DNA double helix into individual strands, was encoded by ORF200, showing 99.8% identity to that of *Vibrio* phage phi-pp2. DNA primase encoded by ORF226 has 100% identity to that of bacteriophage KVP40, which can initiate DNA synthesis. In addition, enzymes involved in nucleotide metabolism including the exonuclease and the endonuclease, were encoded by ORF229 and ORF279, respectively, both showing 100% identity to those of bacteriophage KVP40. These enzymes and proteins were responsible for generating deoxyribonucleotides for phage DNA synthesis by hydrolyzing host genomic DNA and RNA (Gao et al., 2020). In structural protein module, ORF186 was predicted to be a major capsid protein with 100% identity to that of bacteriophage KVP40, and the typical tail structural proteins including tail tube protein, tail sheath protein, and baseplate protein were encoded by ORF180, ORF179, and ORF 163–179, respectively. In the packing protein module, the large terminase subunit was encoded by ORF178 with 100% identity to that of bacteriophage KVP40, and the small terminase subunit was encoded by ORF177 with 100% identity to that of *Vibrio* phage phi-pp2. ORF181 was predicted to encode a portal protein with 100% identity to that of *Vibrio* phage phi-pp2, which can infect DNA into the host cell through a pathway formed by portal protein (Yasui et al., 2017). Just as reported (Paul et al., 1998), terminase plays an important role in the DNA packing processes of the phage genome. The large terminase subunit plays a major role in packaging, while the small terminase subunit binds specifically to the genome and directs the packaging process. The proteins for metabolic and regulated functions were found in ORF18 and ORF237, which encoded thymidylate kinase and thymidylate synthase, respectively, involving in the metabolism of nucleic acids and show100% identity to that of bacteriophage KVP40 and *Vibrio* phage phi-pp2, respectively. In addition, protein rllA, the one affecting or avoiding the lysis of another phage, was encoded by ORF360 with 95.36% identity to that of *Vibrio* phage phi-pp2. It allows the cell infected by the first invader to avoid or exclude the invasion of another phage virion (Moussa et al., 2014).

In the genome of PVA8, the genes for virulence, antibiotic resistance, and lysogeny were not found, suggesting that this phage could be used to control *Vibrio* without much safety concern. According to the analysis using tRNAscan-SE, totally up to 27 tRNAs were found in the genome of PVA8, which was on the high end of tRNA numbers as found in other similar phages genome. The high number of tRNA may benefit the phage in the synthesis of its own proteins (Lu et al., 2020).

**Figure 6:**
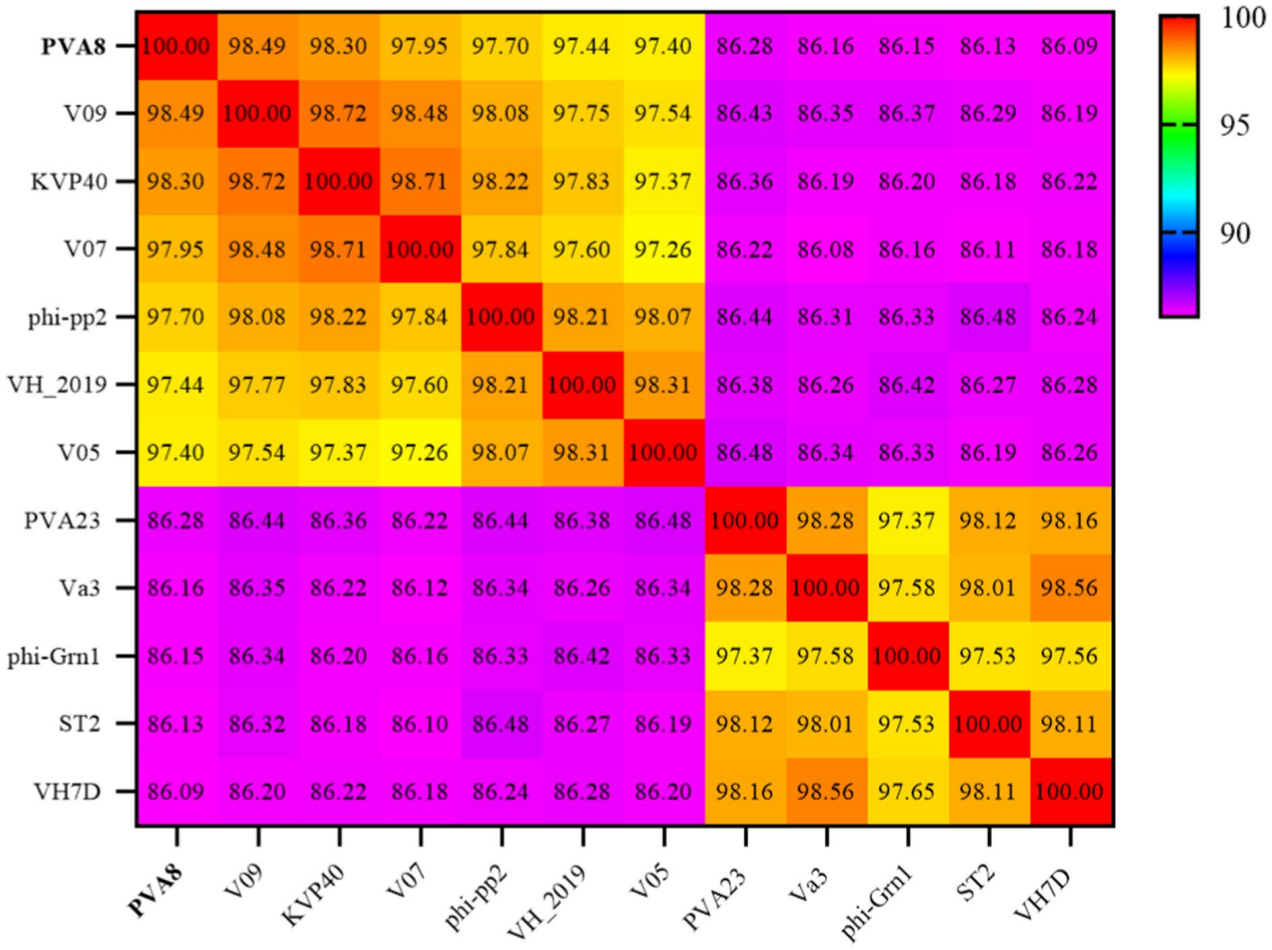
Average nucleotide identity heatmap. The percentage identity values range from 86.09% (purple) to 100% (red).

**Figure 7:**
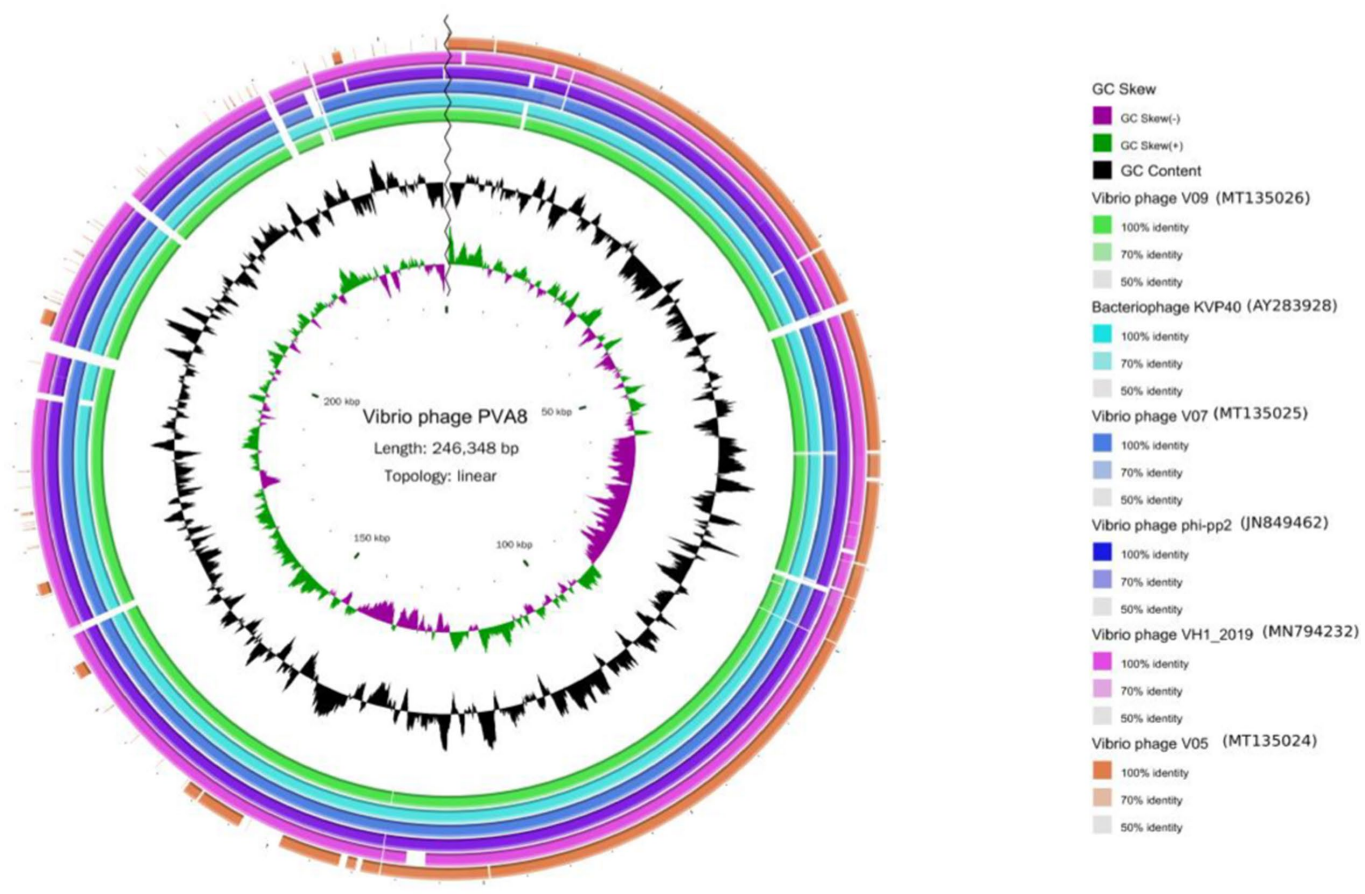
The genomic comparison map between phage PVA8 and six other most similar reference phages. From the outside to the inside: (1) The first circle represents the *Vibrio* phage V05 sample; the second circle represents the *Vibrio* phage VH1_2019 sample; the third circle represents the *Vibrio* phage phi-pp2 sample; the fourth circle represents the *Vibrio* phage V07 sample; the fifth circle represents the bacteriophage KVP40 sample and the sixth circle represents the *Vibrio* phage V09 sample. (2) GC content represented by the seventh circle. (3) G + C skew represented by the eighth circle. (4) The ninth circle is the scale.

The BLASTn comparison result showed that the genome of PVA8 had high nucleotide homology with *V. parahaemolyticus-*infecting bacteriophage KVP40 (98.99% identity, 97% coverage), *V. parahaemolyticus-*infecting phage V09 (98.72% identity, 96% coverage), *V. parahaemolyticus-*infecting V07 (98.71% identity, 96% coverage), *Vibrio natriegens-*infecting phage VH1_2019 (98.48% identity, 95% coverage), *V. parahaemolyticus-*infecting phage V05 (98.17% identity, 92% coverage), and *V. parahaemolyticus-*infecting and *V. alginolyticus*-infecting phage phi-pp2 (98.16 identity, 96% coverage), respectively. By using the ANI heatmap analysis, PVA8 was aligned with a panel of bacteriophages including KVP40, V09, V07, VH1_2019, V05, and phi-pp2, with the ANIm percentage identity each of the phages derived as 98.30%, 98.49%, 97.95%, 97.44%, 97.40%, and 97.70%, respectively (Figure 6). CGView tool was employed to charting comparative map of the whole-genomes of phage PVA8 and the most similar reference phages selected from the NCBI database. As presented in Figures 7, we have used the phage PVA8 as a reference genome and BLAST with the most similar reference phages selected from the NCBI database. The similarities among these phages were plotted by a solid color circle and dissimilarities among the phages were plotted by a white color circle. For the seven phages, high similarities have been observed.

**Figure 8:**
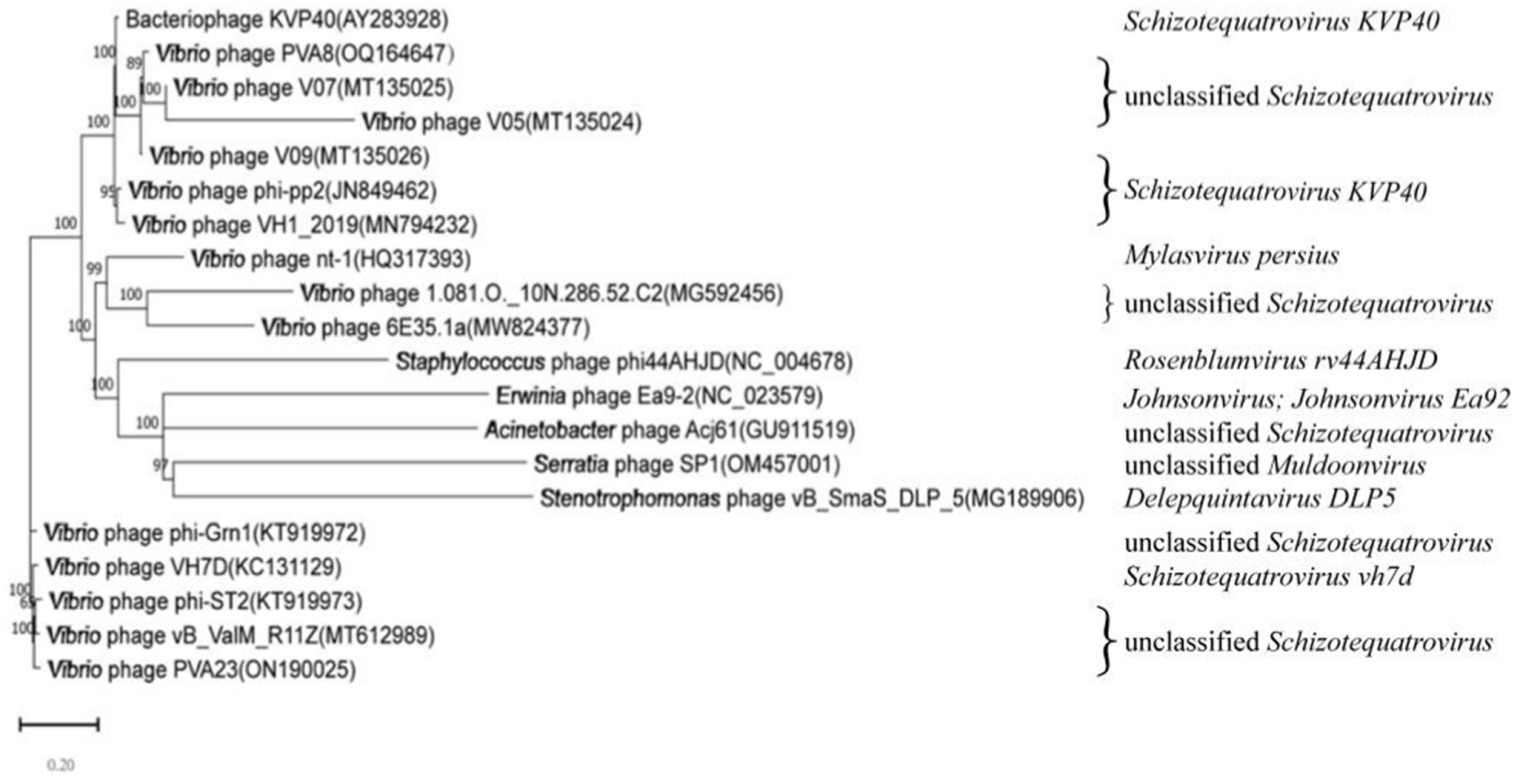
Phylogenetic analysis based on the nucleotide sequence of the complete genome of phage PVA8. The phylogenetic tree was constructed using the Maximum Likelihood method with 1,000 bootstrap replicates.

**Figure 9:**
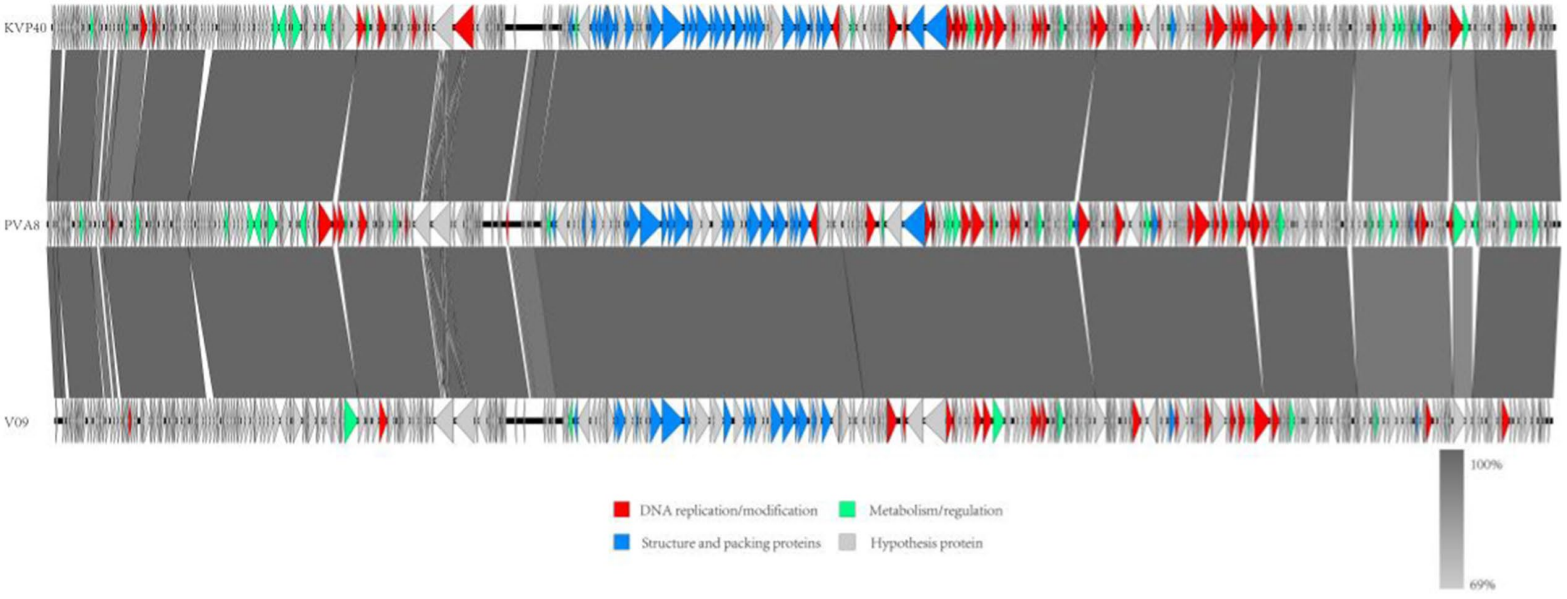
Comparative genome map of a multiple-genome comparison (amino acid level) performed using Easyfig software. Open reading frames (ORFs) are shown as arrows to indicate the direction of transcription and are colored in accordance with their predicted functions: DNA replication/modification (red arrows), structure and packing proteins (blue arrows) and metabolism/regulation (green arrows). Gray arrows represent ORFs of unknown functions (hypothetical protein). Gray shading indicates nucleotide identity between sequences (69%–100%).

In order to decipher the evolutionary relationships of PVA8 with the other closely related homologous phages, sequences of the complete genome of PVA8 were aligned with that of the other closely related phages downloaded from NCBI database. The phylogenetic tree of the complete genome (Figure 8) demonstrated that phage PVA8 was more closely related to V07 and V05, forming a clear clade and showing the homologous coverage with 89%, and following V09 and KVP40. The comparison of whole genomic sequence alignments of PVA8, KVP40, and V09 was conducted, which enabled the depiction of the relationships of the phages at the genomic levels. As aforementioned, the ORFs assigned to basic phage-related functions can be identified and categorized. Therefore, we categorize the genes of PVA8 into five groups: DNA replication/modification, structure and packing proteins, metabolism/regulation, host lysis, and hypothetical protein. Similarly, the genome sequences of KVP40 and V09 were down loaded from NCBI and grouped in the same way. As shown in Figure 9, PVA8 was homologous to KVP40 and V09 in a very big portion of the genome, and the nucleotide identities were both not less than 69%.

### 3.8. Evaluation of phage PVA8 application against *Vibrio parahaemolyticus* VP8 infections in laboratory shrimp culture trial

**Figure 10:**
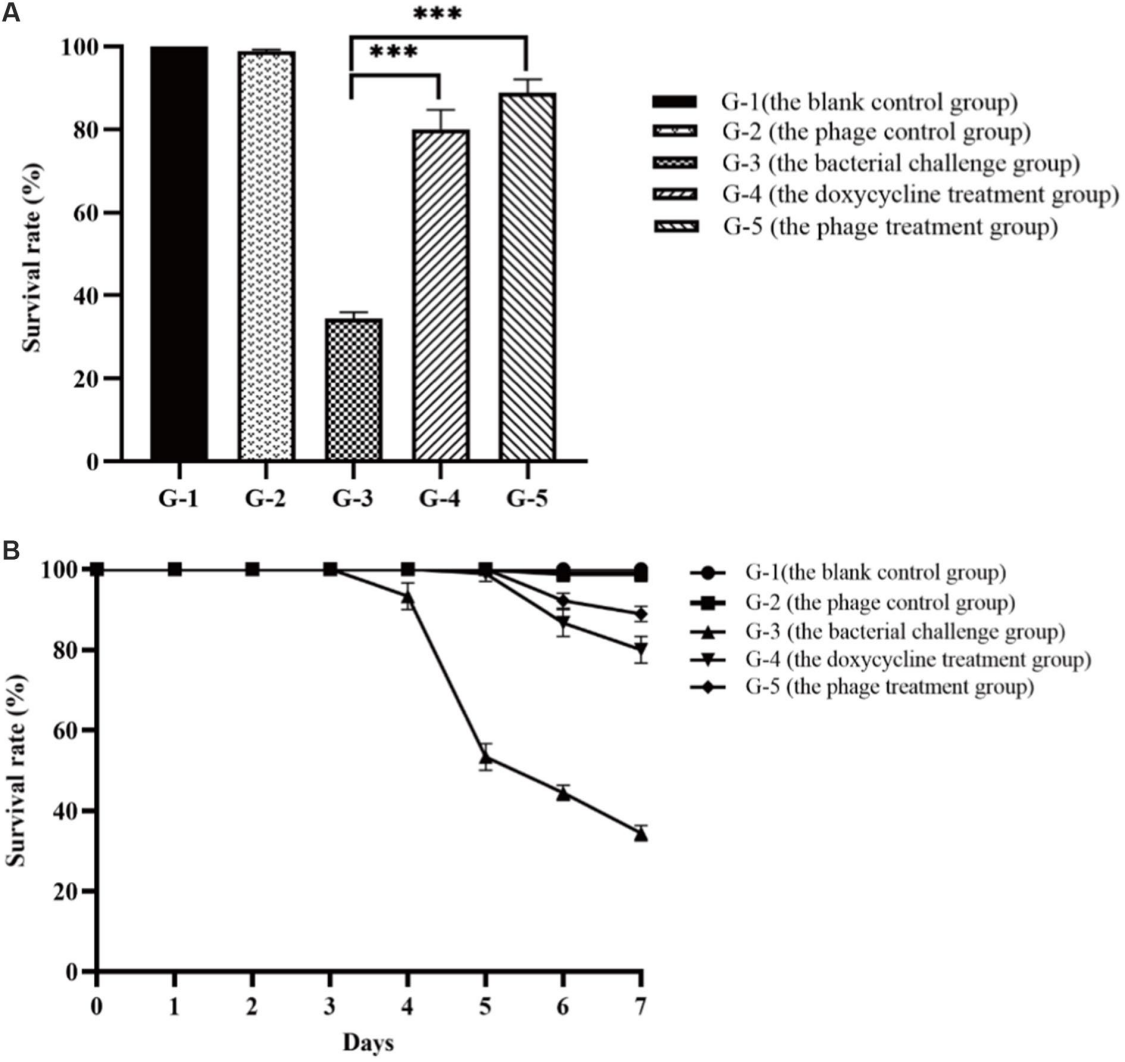
Comparison of the survival rates of the shrimp in different treatment groups. **(A)** Survival rates of the shrimp of each group after the 7-day treatment; **(B)** Survival rates of the shrimp of each group in the experiment duration. G-1: Group 1 was the blank control group without any treatment. G-2: Group 2 received of the phage stock at the final concentration of 2 × 10^7^ PFU/mL. G-3: Group 3 was challenged with *Vibrio parahaemolyticus* strain VP8 at concentration of 2 × 10^6^ CFU/mL. G-4: Group 4 was treated with 10 mg/l doxycycline after two-day challenge of strain VP8 at 2 × 10^6^ CFU/mL. G-5: Group 5 received treatment of the phage stock at the final concentration of 2 × 10^7^ PFU/mL after two-day challenge of strain VP8 at 2 × 10^6^CFU/mL. Asterisks above the columns indicate significant differences at the *p* < 0.01 level.

The laboratory shrimp cultivation trials were conducted to evaluate the effectiveness of phage PVA8 against the pathogenic infections of the shrimp. In the trials, one blank control group and four experimental groups were arranged. It was observed that in the blank group shrimp bodies were transparent, rigid and cyan-black in color, with clearly-edged and brownish black hepatopancreas shaped like an inverted gourd. The survival rate of the shrimp in this group was 100% (G-1 in Figures 10A,B). When the shrimp challenged and infected with VP8, the skin color of the infected shrimp became white and turbid and the body was soft, meanwhile the hepatopancreas was enlarged and light yellow in color. The shrimp survival rate in this group declined to 34.43% in 7 days (G-3 in Figures 10A,B). In comparison, in the group with phage treatment, the shrimp survival rate amounted to 88.89% (G-5 in Figures 10A,B), which was comparable or superior to that of the antibiotics group (80%; G-4 in Figures 10A,B). To exclude the adverse effect of PVA8 to shrimp, phage alone was used for the injection. The survival rate in G-2 was 98.89% (G-2 in Figures 10A,B), very close to that of group 1, suggesting that phage alone does not bring about adverse effects to shrimp.

### 3.9. Evaluation of phage PVA8 application for *Vibrio* control in shrimp farming plant

**Figure 11:**
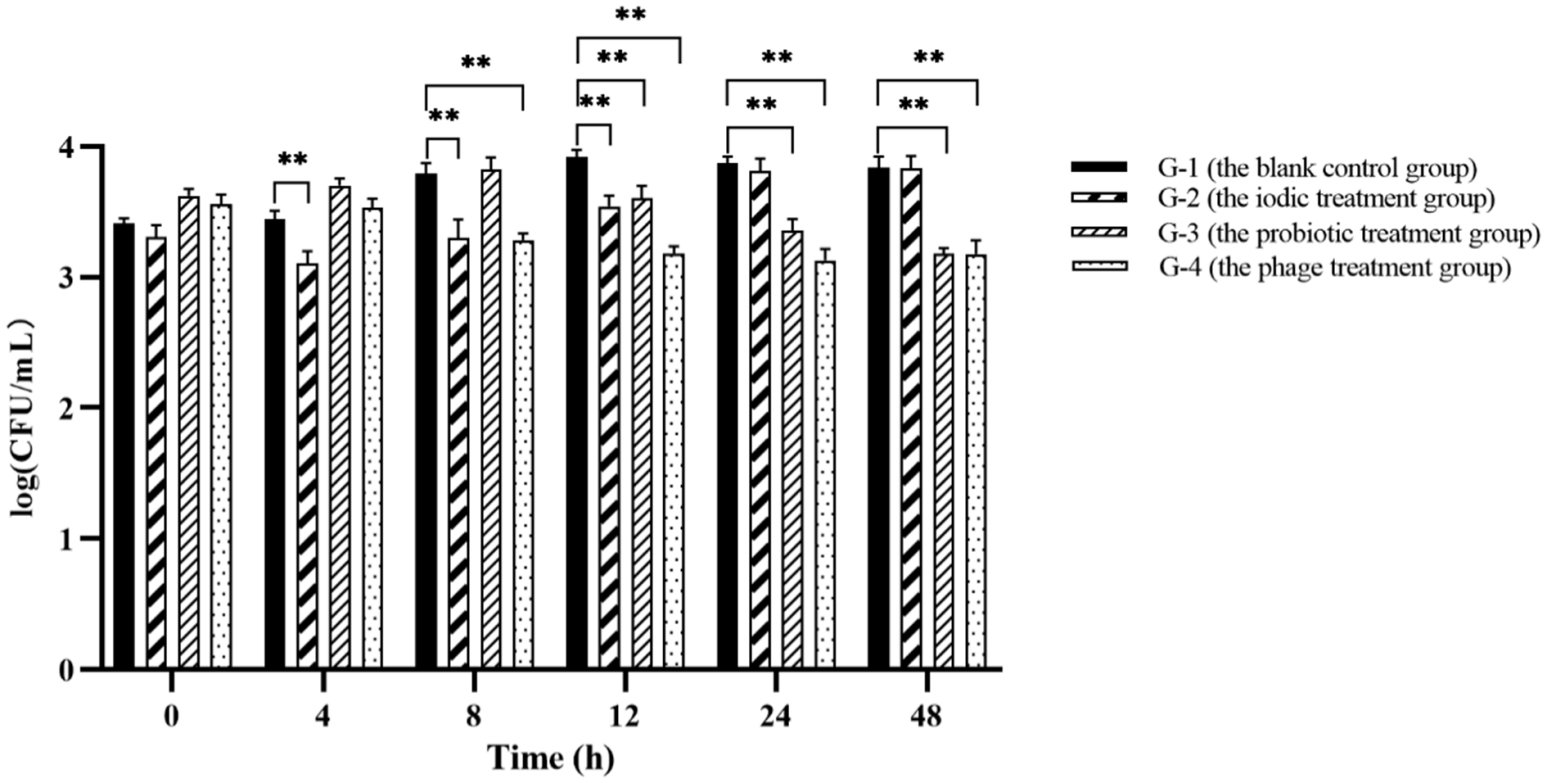
The result of phage PVA8 application in the shrimp farming plant. G-1: Group 1 was the blank control group receiving no control agent. G-2: Group 2 was the positive control group 1 receiving an iodic disinfectant (found in market) at dose of 5 mg/L. G-3: Group 3 was the positive control group 2 receiving a probiotic product (Qingdao Bioantai Biological Technology Co., LTD) at dose of 5 mg/L. G-4: Group 4 was the phage treatment group receiving the phage stock with MOI at 0.1. Asterisks above the columns indicate significant differences at the *p* < 0.01 level.

To evaluate the effectiveness of phage PVA8 in the commercial shrimp farming practice, application trials were designed. In the trials, one blank control group and three experimental groups were included. It was found that *Vibrio* content in each blank control kept constant or increasing with time (G-1 in Figure 11), which reflected the challenge presented by *Vibrio* if no intervention applied. The group 2 that received the treatment of an iodic disinfectant showed valid effect at 4 h, but then the *Vibrio* kept bouncing and elevated to the same level as in the control at 24 h (G-2 in Figure 11). The application of the probiotic product showed obvious effect at 12 h and kept constant decrease after that (G-3 in Figure 11). In comparison, the *Vibrio* in the ponds with phages maintained a tendency of decreasing during the whole trial (G-4 in Figure 11). These results of phage PVA8 against the *Vibrio*, especially *V. alginolyticus* and *V. parahaemolyticus* for specifically, in farming plant trials confirmed that the phage could serve as an effective biological agent for the control of *Vibrio* in farming plant practice.

## 4. Discussion

The emergence of antibiotic-resistant pathogens and the awareness of the adverse effects of antibiotics to animals have necessitated alternatives to antibiotics used in aquaculture (Wang et al., 2017). Phages have long been regarded as the safe yet effective substitute to antibiotics used in aquaculture. Phages exist in natural aquatic environments, serving as native antibacterial agents that maintain the balance of ecosystem. There are many cases with exemplified the success of phage therapy against *Vibrio* species (Mateus et al., 2014). Generally, in order to screen a truly useful bacteriophage, a panel of crucial factors need to be taken into considerations, such as its effectiveness against the pathogens that found in real farming practice, its safety for use as well as its feasibility for engineering manipulations.

*Vibrio alginolyticus* and *V. alginolyticus* are the two most important opportunistic pathogens that infect a variety of aquatic animals, such as fish, shellfish, and shrimp, causing mass mortality cases and considerable economic losses (Letchumanan et al., 2016; Sasikala and Srinivasan, 2016). They are also among the major foodborne pathogens in seafood that very often bring about severe risks to the health of consumers (Gao et al., 2020). The main virulence factors of *V. alginolyticus* include extracellular products (alkaline serine protease, collagenase), outer membrane proteins, hemolysins, ferric uptake system and virulence expression regulation gene system (Fethi et al., 2011). *Vibrio parahaemolyticus* is one of the major cause of disease, mortality, and economical losses in the shrimp aquaculture industry (Lomelí-Ortega and Martínez-Díaz, 2014). It can cause AHPND, one of the most serious diseases in shrimp aquaculture, resulting in a near 100% mortality rate within a week after the first symptoms appearing in cultured shrimp (Lai et al., 2015). The causative agents of the disease have been identified as two *Pir*-like toxins, *PirA* and *PirB*, which are encoded by a plasmid (Lee et al., 2015).

The purpose of this work is to identify the potential phages that could be used for the control of two pathogenic *Vibrio*, *V. alginolyticus* and *V. alginolyticus*. In order to obtain the real virulent *Vibrio*, a panel of *Vibrio* was isolated and selected. A group of common virulence genes including alkaline serine protease gene (*AspA*), ferric uptake system gene (*fur*), and hemolysin genes (*tlh*, *tdh,* and *trh*) of *V. alginolyticus* and photorhabdus insect related toxin A gene (*PirA*), virulence-regulating genes (*toxR*), and hemolysin genes (*tlh*, *tdh,* and *trh*) of *V. parahaemolyticus* were used for selecting the pathogenic *V. alginolyticus*. Among the isolates, two strains, *V. alginolyticus* VA15 and VA17, were found carrying all the five virulence genes, thus they were used as the host for the followed phage screening. Similarly, the signature genes for *V. parahaemolyticus* (Han et al., 2015), *toxR* and *tlh,* were applied for search *V. parahaemolyticus* in general, whereas the gene causative to the AHPND disease (Lee et al., 2015), *PirA*, was used for obtain the virulent *V. parahaemolyticus* in specific. Among the isolates of *V. parahaemolyticus* strains, VP5, VP7, VP8, and VP15, were found carrying the *PirA* virulence gene, thus they were also used as the host for phage screening.

Through screening against the six pathogenic *Vibrio* host, a potent phage PVA8 was identified. It was isolated from the shrimp farming sewage samples containing *V. alginolyticus*. The bacteriophage belongs to the *Straboviridae* family with a double-stranded DNA genome sequence in length of 246,348 bp, a prolate icosahedral capsid, and a long contractile tail with associated baseplate and extended tail fibers. Subsequent genome analysis showed that neither toxic genes nor antibiotic resistant genes identified in the genome of PVA8, which supposed its low safety concern for use.

The results of the BLASTn, genomic comparison and the phylogenetic tree based on the complete genome all showed that PVA8 had highest nucleotide homology with bacteriophage KVP40 and *Vibrio* phage V09. All the three phages are giant phages with linear dsDNA and large genome sizes, and are all belonged to the *Straboviridae* family with same morphological characteristics. Both of PVA8 and KVP40 are broad host range phages, different with V09 which is specific for *V. parahaemolyticus.* PVA8 could infect at least seven *Vibrio*, comparable to KVP40 that infect at least eight *Vibrio* plus one *Photobacterium* species.

The three phages all encoded high numbers of tRNAs with 27 (PVA8), 30 (KVP40), and 27 (V09), respectively. The tRNAs as translation-related genes contributing in the phage infection process and the synthesis of phage capsid and tail proteins are usually found in phage genomes (Mei and Zhu, 2015). In general, virulent phages contain more tRNAs than lysogenic phages, the reason might be that there is a significant association between tRNA distribution and codon usage which leading to an enhanced translational efficiency (Li et al., 2012). The high number of tRNA might benefit the phage in synthesis of its own proteins, compared with other phages that encoded fewer tRNAs, which mainly depend of the host translation machinery. Therefore, PVA8 was considered as a strong virulent phage (Lu et al., 2020).

It is noteworthy that phage PVA8 and bacteriophage KVP40 are alike in a panel of aspects, including phenotypic characteristics, similarities in genomic features, broad host ranges, etc. The tail is an important component of the phage, and its adsorption devices specialize in identifying and binding receptors on the surface of bacteria, initiating virus infection and DNA transferring. The complexity of this devices varies from single tail fiber to complex tail tip combination or complex base plate (Ding et al., 2020). Interestingly, it was found that the annotation information of most tail proteins of phage PVA8 have 100% similarity to those found in bacteriophage KVP40. The resemblance in tail devices may partial reason why the broad host range nature of the two phages.

For double-stranded DNA bacteriophages, the bacterial lysis is achieved by the phage-encoded muralytic enzyme called endolysins that hydrolyze the peptidoglycan (PG) layer of the host bacterial cell wall during the final stage of the phage reproduction events (O'Flaherty et al., 2009). There are three different mechanisms explaining the lysis events by endolysins. The most explicitly demonstrated mechanism evolves a lytic system called holin-endolysin, it involves a second phage-encoded protein called holin to assist endolysin acts on PG layer (Matamp and Bhat, 2019). Holin allows endolysin to diffuse through pores in the membrane and target the PG layer by depolarizing the cytoplasmic membrane, as most of endolysins lack the signal peptide sequence and cannot cross the cell membrane (Matamp and Bhat, 2019). Based on the mechanism of action, endolysins have at least four different hydrolase activities that degrade the cell wall, which are glycoside transferase, lysozyme, amidase and endopeptidase, respectively (Matamp and Bhat, 2019). These four hydrolase activities can all be found in the endolysins of *Vibrio* phages, especially glycoside transferase (Misol et al., 2020). However, none of endolysins mentioned above was found in the annotation results of the PVA8 genome. Similarly, neither endolysins were identified in bacteriophage KVP40. For bacteriophage KVP40, the presence of several copies of genes encoding proteins linked with phage tail or tail fibers in the genome suggest an increased flexibility in host range adaptation (Miller et al., 2003). Thus, here we suppose that similar host range adaptation might also happen to phage PVA8. Further evidences could be furnished in the future via the comparison of the tail-related genes as used elsewhere (Jacobs-Sera et al., 2012).

In this work, we succeeded in evaluating the preparation of phage PVA8 for the use against *Vibrio* pathogens in aquatic farming practice, with therapeutic and prophylactic of *Vibrio* infections trials arranged. After phage treatment, the shrimp survival rate in control was improved to 88.89% (from 34.43% in the control receiving *Vibrio* only), which is comparable to the best result reported elsewhere (Ding et al., 2020; Wu et al., 2021). It was noteworthy that preparation of phage PVA8 outperformed the chemical disinfectant in *Vibrio* control, and it even achieve faster inhibition of the pathogenic *+*than that of the best probiotic product currently used in the market. Therefore, we suggested that PVA8 based phage therapy might be a potential alternative to antibiotics regarding the control of pathogenic *Vibrio* in aquaculture (Stalin and Srinivasan, 2017).

In this study, a potential *Vibrio* lytic bacteriophage PVA8 with broad host ranges was screened and characterized. The phage was belonged to the *Straboviridae* family with a high lytic capability. It demonstrated a panel of features like broad host ranges, low MOI, large burst size, and decent tolerances to adverse conditions, which are promising merits for antibiotics substitution. Genome analysis revealed that there was neither virulence nor antibiotic resistance genes in the genome, further convincing its environmental-friendly nature and safety for application. The anti-*Vibrio* potency of the phage was indicated by lab shrimp trials and was further proved by the pond application. The fact that PVA8 could rapidly yet effectively reduce the level of *Vibrio* in pond practice doubtlessly provided evidence that PVA8 might serve as a biological agent for *Vibrio* control in aquaculture farming prophylactically and therapeutically.

## Data availability statement

The datasets presented in this study can be found in online repositories. The names of the repository/repositories and accession number(s) can be found in the article/Supplementary material.

## Author contributions

ZH and JF conceived and designed the study. JF and YL performed the experiments. JF, LZ, and CW analyzed the data. JF and ZH wrote the paper. ZH managed the project. All authors contributed to the article and approved the submitted version.

## Funding

This research was funded by Special Fund for TaiShan Industrial Experts Program (tscy202006017) and Science and Technology Projects in Key Areas of Guangdong (2020B0202010001).

## Conflict of interest

JF and ZH were employed by Marine Biomedical Research Institute of Qingdao Co., Ltd. JF, CW, and ZH were employed by Qingdao Bioantai Biotechnology Co., Ltd.

The remaining authors declare that the research was conducted in the absence of any commercial or financial relationships that could be construed as a potential conflict of interest.

## Publisher’s note

All claims expressed in this article are solely those of the authors and do not necessarily represent those of their affiliated organizations, or those of the publisher, the editors and the reviewers. Any product that may be evaluated in this article, or claim that may be made by its manufacturer, is not guaranteed or endorsed by the publisher.

## Footnotes

^1^ http://trna.ucsc.edu/tRNAscan-SE/

^2^ http://www.mgc.ac.cn/VFs/main.htm

^3^ https://card.mcmaster.ca/analyze/rgi

^4^ http://www.microbesonline.org/fasttree/
