# Supplementary material for: Characterization of vB_ValM_PVA8, a broad-host-range bacteriophage infecting Vibrio alginolyticus and Vibrio parahaemolyticus
Source: Front Microbiol. 2023 May 12;14:1105924. doi: 10.3389/fmicb.2023.1105924 (PMC10213691; doi:10.3389/fmicb.2023.1105924)
Supplement: Supplementary file 1 [file Data_Sheet_1.pdf]

TABLE S1 | Phage isolation results using the *V. alginolyticus* strains as hosts.

Number	Phage	Plaque morphology	The host *V. alginolyticus*	The lysis rate (%)
1	PVA1	Small and bright	VA0	25% (10/40)
2	PVA2	Small and bright	VA1	15% (6/40)
3	PVA3	Big and fuzzy	VA3	22.5% (9/40)
4	PVA4	Small and bright	VA5	5% (2/40)
5	PVA5	Small and bright	VA7	12.5% (5/40)
6	PVA6	Big and bright	VA9	37.5% (15/40)
7	PVA7	Small and bright	VA9	27.5% (11/40)
8	PVA8	Small and bright	VA10	82.5% (33/40)
9	PVA9	Big and bright	VA11	50% (20/40)
10	PVA10	Small and bright	VA11	7.5% (3/40)
11	PVA11	Small and bright	VA13	22.5% (9/40)
12	PVA12	Small and bright	VA13	5% (2/40)
13	PVA13	Small and bright	VA14	35% (14/40)
14	PVA14	Small and bright	VA15	17.5% (7/40)
15	PVA15	Big and fuzzy	VA15	27.5% (11/40)
16	PVA16	Small and bright	VA15	2.5% (1/40)
17	PVA17	Small and bright	VA16	32.5% (13/40)
18	PVA18	Small and fuzzy	VA17	5% (2/40)
19	PVA19	Small and bright	VA20	20% (8/40)
20	PVA20	Small and bright	VA6	10% (4/40)
21	PVA21	Big and fuzzy	VA9	42.5% (17/40)
22	PVA22	small and bright	VA10	35.5% (13/40)
23	PVA23	Small and bright	VA11	65% (26/40)
24	PVA24	Small and fuzzy	VA15	15% (6/40)
25	PVA25	Small and bright	VA15	30% (12/40)
26	PVA26	Small and bright	VA0	22.5% (9/40)
27	PVA27	Small and bright	VA6	5% (2/40)
28	PVA28	Big and bright	VA7	30% (12/40)
29	PVA29	Small and bright	VA7	50% (20/40)
30	PVA30	Small and bright	VA13	12.5% (5/40)
31	PVA31	Small and bright	VA21	10% (4/40)
32	PVA32	Small and fuzzy	VA22	17.5% (7/40)
33	PVA33	Small and bright	VA22	35% (14/40)
34	PVA34	Small and fuzzy	VA22	25% (10/40)
35	PVA35	Small and bright	VA23	20% (8/40)
36	PVA36	Big and bright	VA24	40% (16/40)
37	PVA37	Small and bright	VA26	22.5% (9/40)
38	PVA38	Small and bright	VA26	7.5% (3/40)
39	PVA39	Big and fuzzy	VA30	42.5% (17/40)
40	PVA40	Small and fuzzy	VA31	10% (4/40)
41	PVA41	Big and bright	VA31	57.5% (23/40)

42	PVA42	Big and fuzzy	VA34	32.5% (13/40)
43	PVA43	Big and bright	VA36	27.5% (11/40)
44	PVA44	Small and bright	VA27	17.5% (7/40)
45	PVA45	Small and bright	VA40	20% (8/40)
46	PVA46	Small and bright	VA40	10% (4/40)
47	PVA47	Big and bright	VA41	27.5% (11/40)
48	PVA48	Small and bright	VA41	15% (6/40)
49	PVA49	Big and bright	VA22	55% (22/40)
50	PVA50	Small and bright	VA23	35% (14/40)
51	PVA51	Small and fuzzy	VA28	5% (2/40)
52	PVA52	Small and bright	VA30	15% (6/40)
53	PVA53	Big and fuzzy	VA1	45% (18/40)
54	PVA54	Small and bright	VA1	17.5% (7/40)
55	PVA55	Small and bright	VA7	7.5% (3/40)
56	PVA56	Small and bright	VA9	27.5% (11/40)
57	PVA57	Big and bright	VA8	45% (18/40)
58	PVA58	Small and bright	VA15	35% (14/40)
59	PVA59	Small and bright	VA11	22.5% (9/40)
60	PVA60	Big and fuzzy	VA11	50% (20/40)
61	PVA61	Small and bright	VA10	25% (10/40)
62	PVA62	Small and bright	VA14	15% (6/40)
63	PVA63	Small and bright	VA19	17.5% (7/40)

TABLE S2 | Phage isolation results using the *V. parahaemolyticus* strains as hosts.

Number	Phage	Plaque morphology	The host *V. parahaemolyticus*	The lysis rate (%)
1	PVP1	Small and bright	17802	10.71% (3/28)
2	PVP2	Small and bright	VP1	21.43% (6/28)
3	PVP3	Small and bright	VP3	25% (7/28)
4	PVP4	Big and fuzzy	VP2	35.71% (10/28)
5	PVP5	Small and fuzzy	VP5	7.14% (2/28)
6	PVP6	Small and fuzzy	VP6	32.14% (9/28)
7	PVP7	Small and fuzzy	VP6	39.29% (11/28)
8	PVP8	Small and fuzzy	VP7	28.57% (8/28)
9	PVP9	Big and fuzzy	VP7	14.29% (4/28)
10	PVP10	Big and bright	VP8	7.14% (2/28)
11	PVP11	Small and fuzzy	VP9	35.71% (10/28)
12	PVP12	Small and bright	VP9	21.43% (6/28)
13	PVP13	Small and bright	VP10	32.14% (9/28)
14	PVP14	Small and bright	VP11	42.86% (12/28)
15	PVP15	Small and bright	VP12	35.71% (10/28)
16	PVP16	Small and bright	VP13	14.29% (4/28)
17	PVP17	Small and bright	VP15	39.29% (11/28)
18	PVP18	Small and bright	VP16	25% (7/28)
19	PVP19	Small and bright	VP19	3.57% (1/28)

20	PVP20	Small and fuzzy	VP20	10.71% (3/28)
21	PVP21	Big and fuzzy	VP2	28.57% (8/28)
22	PVP22	Small and fuzzy	VP4	39.29% (11/28)
23	PVP23	Small and fuzzy	VP7	14.29% (4/28)
24	PVP24	Small and fuzzy	VP8	53.57% (15/28)
25	PVP25	Big and fuzzy	VP9	21.43% (6/28)
26	PVP26	Big and bright	VP13	32.14% (9/28)
27	PVP27	Small and fuzzy	VP19	25% (7/28)
28	PVP28	Big and fuzzy	VP6	21.42% (6/28)
29	PVP29	Small and bright	VP9	14.29% (4/28)
30	PVP30	Small and fuzzy	VP11	% (12/28)
31	PVP31	Small and bright	VP18	10.71% (3/28)
32	PVP32	Small and fuzzy	VP20	28.57% (8/28)
33	PVP33	Small and bright	VP22	25% (7/28)
34	PVP34	Small and fuzzy	VP21	14.29% (4/28)
35	PVP35	Small and bright	VP27	28.57% (8/28)
36	PVP36	Small and fuzzy	VP27	32.14% (9/28)
37	PVP37	Small and fuzzy	VP32	46.43% (13/28)
38	PVP38	Small and fuzzy	VP33	32.14% (9/28)
39	PVP39	Small and fuzzy	VP34	38.29% (11/28)
40	PVP40	Small and fuzzy	VP34	10.71% (3/28)
41	PVP41	Small and fuzzy	VP35	35.71% (10/28)
42	PVP42	Small and bright	VP36	25 % (7/28)
43	PVP43	Small and bright	VP37	21.42% (6/28)
44	PVP44	Small and fuzzy	VP37	7.14% (2/28)
45	PVP45	Small and fuzzy	VP38	32.14% (9/28)
46	PVP46	Small and fuzzy	VP39	35.71% (10/28)
47	PVP47	Small and fuzzy	VP39	35.71% (10/28)
48	PVP48	Small and fuzzy	VP15	28.57% (8/28)
49	PVP49	Small and fuzzy	VP16	10.71% (3/28)
50	PVP50	Small and bright	VP5	39.29% (11/28)
51	PVP51	Small and fuzzy	VP3	17.86% (5/28)
52	PVP52	Small and fuzzy	VP1	32.14% (9/28)
53	PVP53	Small and bright	VP8	21.42% (6/28)
54	PVP54	Small and bright	VP7	21.42% (6/28)
55	PVP55	Small and bright	VP19	35.71% (1/28)
56	PVP56	Small and bright	VP20	28.57% (8/28)
57	PVP57	Big and fuzzy	VP21	25% (7/28)
58	PVP58	Big and fuzzy	VP33	10.71% (3/28)
59	PVP59	Small and bright	VP35	39.29% (11/28)
60	PVP60	Small and fuzzy	VP39	32.14% (9/28)
61	PVP61	Small and fuzzy	VP38	25% (7/28)
